# Yixintai granules enhance glucose and lipid metabolism after ischemic heart failure by regulating inflammatory factors via the LPS/TLR4/NF-κB signaling pathway

**DOI:** 10.3389/fimmu.2026.1984202

**Published:** 2026-10-16

**Authors:** Ziyan Wang, Chengxin Liu, Jiaming Wei, Yiwen Mao, Hui Yuan, Min Shi, Ruifang Lin, Zehui Ding, Yinli Zhou, Zhihua Guo, Jinwen Ge, Yun Long, Ya Li

**Affiliations:** 1 Cardiovascular Medicine Department, The First Hospital of Hunan University of Chinese Medicine, Changsha, China; 2 Hunan Key Laboratory of Colleges and Universities of Intelligent Traditional Chinese Medicine Diagnosis and Preventive Treatment of Chronic Diseases of Hunan Universities of Chinese Medicine, Hunan University of Chinese Medicine, Changsha, China; 3 Department of Critical Care Medicine, The Second Hospital of Hunan University of Chinese Medicine, Changsha, China; 4 School of Traditional Chinese Medicine, Hunan University of Chinese Medicine, Changsha, China; 5 Department of Chinese Medicine, Hunan Aerospace Hospital, The Affiliated Aerospace Hospital of Hunan Normal University, Changsha, China; 6 Hunan Academy of Traditional Chinese Medicine, Changsha, China; 7 School of Pharmacy, Hunan University of Chinese Medicine, Changsha, China

**Keywords:** glucose and lipid metabolism, inflammatory factors, ischemic heart failure, LPS/TLR4/NF-κB signaling pathway, Yixintai granules

## Abstract

**Background:**

Ischemic heart failure (IHF) severely impacts patients’ quality of life and leads to frequent hospitalizations, increased healthcare costs, and increased mortality rates, thus posing a substantial global health challenge. Myocardial ischemia is a primary trigger of HF. Yixintai granules (YXT), a traditional Chinese medicine (TCM) formulation, have demonstrated clinical benefits in HF treatment; however, their underlying mechanisms remain unclear.

**Methods:**

The components of YXT absorbed into the blood were identified using ultra-high-performance liquid chromatography coupled with mass spectrometry (UHPLC-MS/MS). The key regulatory mechanisms underlying its therapeutic effects on HF were clarified using network pharmacology, and the binding affinity of key YXT components to proteins associated with the LPS/TLR4/NF-κB pathway was assessed through molecular docking. *In vivo* experiments involved the establishment of an HF rat model through the ligation of the left anterior descending coronary artery. *In vitro* experiments utilized an H9C2 cell injury model induced by lipopolysaccharide (LPS), which was subsequently treated with YXT-containing serum.

**Results:**

UHPLC-MS/MS analysis identified 2,581 active components in the YXT and 1,055 active components in the YXT-containing serum. Network pharmacology indicated that the expression of pro-inflammatory cytokines mediated by the LPS/TLR4/NF-κB pathway and glucolipid metabolic disorders may significantly contribute to the anti-HF effects of YXT. Subsequent *in vivo* experiments revealed that YXT enhanced cardiac function, reduced myocardial fibrosis, and improved mitochondrial morphology in HF rats. Additionally, it lowered inflammatory cytokine levels and was associated with altered glucose and lipid metabolic profiles and inhibition of the LPS/TLR4/NF-κB signaling pathway. *In vitro* experiments revealed that YXT-containing serum alleviated LPS-induced H9c2 cardiac cell injury, reduced reactive oxygen species and inflammatory cytokine levels, and was associated with altered glucose and lipid metabolic profiles and inhibition of the LPS/TLR4/NF-κB signaling pathway.

**Conclusion:**

YXT improved cardiac function and attenuated fibrosis and ultrastructural damage in IHF rats, with effects associated with LPS/TLR4/NF-κB signaling inhibition, reduced inflammation, and altered glucose/lipid metabolic profiles.

## Graphical Abstract

**Figure:**
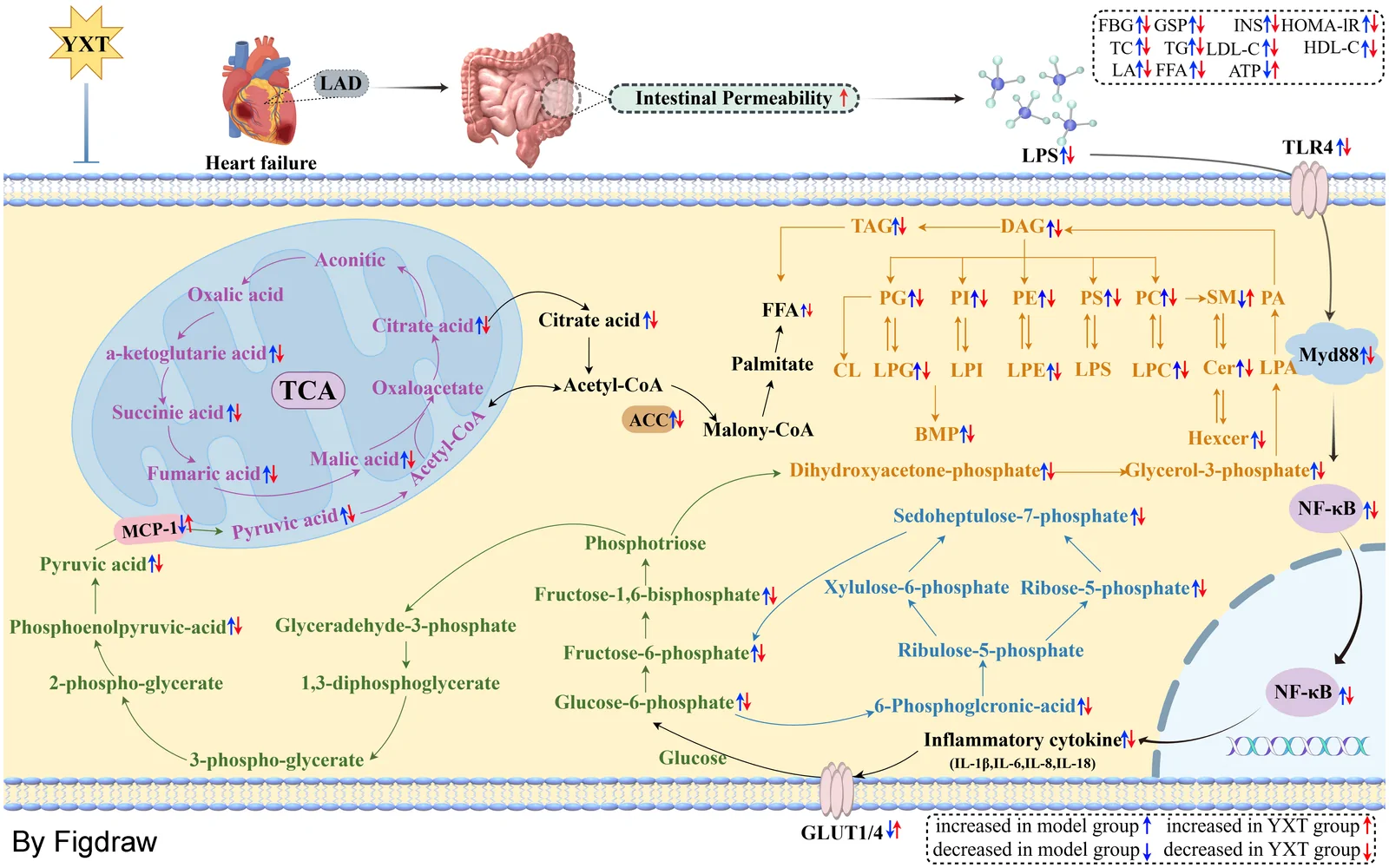

## Introduction

Heart failure (HF) is a complex clinical condition characterized by diverse structural or functional abnormalities of the heart that hinder ventricular pumping and/or filling capacity. HF is a leading cause of death among patients with cardiovascular diseases (1). It is estimated that over 56 million individuals worldwide are affected by HF, which severely compromises patients’ quality of life, results in frequent hospitalizations, and increases healthcare costs and mortality rates, thereby constituting a major global health concern (2). Myocardial ischemia is a primary trigger of HF, inducing damage to cardiac cells within the ischemic region. This damage results in structural and functional cardiac abnormalities that impair both diastolic and systolic function, progressing to ischemic heart failure (IHF) (3).

Treatment strategies for heart failure encompass lifestyle modifications, medications such as beta blockers, ACE inhibitors, ARNIs, and MRAs, and potentially surgery or devices. Diuretics, like Furosemide, are a cornerstone for acute heart failure treatment, and aerobic exercise training is a recognized non-pharmacological approach to improve various aspects of the condition (4–6).Although percutaneous coronary intervention combined with antiplatelet therapy markedly improves survival rates in patients experiencing acute myocardial ischemia, its efficacy in treating post-infarction HF remains limited (7). Therefore, developing novel therapeutic strategies for IHF is essential.

Accumulating evidence has demonstrated that metabolic disturbances in substances, including glucose and fatty acids (FAs) in cardiac cells, leading to changes in energy metabolism pathways, are crucial in HF pathogenesis (8). During cardiac remodeling, the heart’s primary energy substrate switches from fatty acid oxidation to glucose metabolism, which acts as an oxygen-sparing strategy. Alongside this metabolic switch, defects in the tricarboxylic acid (TCA) cycle, oxidative phosphorylation, and high-energy phosphate metabolism collectively contribute to adenosine triphosphate (ATP) depletion. Consequently, these abnormalities reduce contractile function in the failing heart (9). Pathological conditions, including pressure overload, myocardial ischemia, dilated cardiomyopathy, and volume overload, can induce energy metabolism disorders. Pharmacological interventions aimed at ameliorating energy and metabolic deficits have thus emerged as novel strategies to enhance cardiac efficiency (10–12).

Accumulating evidence has demonstrated a significant association between LPS/TLR4/NF-κB signaling pathway and HF pathogenesis. Lipopolysaccharide (LPS), or endotoxin, is a constituent of the outer membrane of many Gram-negative bacteria and serves as a critical molecule in triggering local and systemic inflammatory responses in the host (13). During HF, changes in the gut microbiota result in compromised intestinal barrier permeability, permitting the entry of gut microbiota-derived metabolites, including LPS, into the systemic circulation, exacerbating the systemic inflammatory response in patients with HF (14, 15). Upon binding to toll-like receptor 4 (TLR4) expressed on cardiac cells, lipopolysaccharide (LPS) recruits the adaptor protein MyD88, which subsequently triggers the activation of NF-κB and promotes its nuclear translocation. The TLR4/NF-κB signaling axis plays a key role in the pathogenesis and modulation of inflammatory cardiovascular disorders. In resting conditions, the NF-κB–IκB trimeric complex is sequestered in the cytoplasm. Extracellular stimulation triggers signaling cascades that result in the secretion of pro-inflammatory cytokines and the phosphorylation and degradation of IκB proteins. This process subsequently activates the two subunits of NF-κB, p65 and p50, leading to an increased expression of downstream pro-inflammatory cytokines, including tumor necrosis factor-α (TNF-α), interleukin-1β (IL-1β), IL-6, and IL-18. These cytokines act as endogenous inflammatory inducers by interacting with receptors on target cells (16), leading to inflammatory injury in cardiac cells and facilitating the progression of HF (17).

Traditional Chinese medicine (TCM) has demonstrated substantial efficacy in alleviating symptoms, decreasing hospitalization rates, and reducing mortality among patients with HF (18). Consequently, utilizing the distinctive diagnostic and therapeutic benefits of TCM is crucial for HF prevention and treatment. Yixintai granules (YXT) are a commonly used TCM formula for the clinical management of HF. YXT has been employed for nearly two decades in The First and Second Hospitals of Hunan University of Chinese Medicine. YXT consists of *Astragalus membranaceus (Fisch.) Bunge* (Huangqi), *Panax ginseng C. A. Mey* (Renshen), *Salvia miltiorrhiza Bunge* (Danshen), *Carthamus tinctorius L.* (Honghua), *Polyporus umbellatus (Pers.) Fr* (Fuling), *Poria cocos (Schw.) Wol* (Zhuling), *Alisma plantago-aquatica L.* (Zexie), and *Draba nemorosa L.* (Tinglizi). Its therapeutic effects include the replenishment of qi, activation of blood circulation, promotion of diuresis, and reduction of edema. *In vivo* experiments previously conducted by our research group revealed that YXT can ameliorate myocardial hypertrophy and fibrosis in rats with HF by modulating the TMAO/PKC/NF-κB pathway. Furthermore, it improves myocardial mitochondrial ultrastructure and decreases the concentration of inflammatory factors (19). Based on these preliminary findings and integrating recent advances in HF research, we hypothesize that YXT may regulate glucose and lipid metabolism through the LPS/TLR4/NF-κB pathway, potentially enhancing energy metabolism remodeling in myocardial tissue and contributing to HF treatment. Herein, we utilized a rat model of post-myocardial infarction HF and an LPS-induced H9c2 cell injury model to investigate the regulatory effects of YXT on the LPS/TLR4/NF-κB pathway and on glucose and lipid metabolism. This study aimed to clarify the pharmacological effects of YXT and investigate, at multiple levels, the mechanisms by which YXT regulates glucose and lipid metabolism through the LPS/TLR4/NF-κB pathway, enhances energy metabolism remodeling in IHF myocardial tissue, and prevents and treats HF to offer new insights and guidance for TCM-based prevention and HF treatment.

## Materials and methods

### Experimental animals

We obtained 120 healthy adult male specific pathogen-free (SPF)-grade Sprague-Dawley rats, weighing 200–220 g, from Hunan Silaike Experimental Animal Co., Ltd. (Certificate: No. 430727221102192217). The animals underwent a 3-day acclimatization period to laboratory conditions before the experiment commenced. Standard polypropylene cages were used for rat housing under controlled environmental conditions, including a temperature of 23 ± 2 °C and a relative humidity of 56 ± 4 %, with food and water provided ad libitum. The experimental protocol was approved by the Animal Experiment Ethics Committee of Hunan University of Chinese Medicine (Approval: No. LL2022030207).

### Experimental drugs

YXT is a compound formulation composed of eight Chinese herbal medicines (**Table 1**). All components in TCM granules were processed and prepared at EFONG Pharmaceutical Co., Ltd. (Guangdong, China).

**Table 1:** Composition of YXT.

Chinese name	Botanical name	Dosage(g)
Huangqi	*Astragalus membranaceus* (Fisch.) Bunge	6
Danshen	*Salvia miltiorrhiza* Bunge	2.68
Honghua	*Carthamus tinctorius* L.	0.79
Renshen	*Panax ginseng* C. A. Mey	2
Zexie	*Alisma plantago-aquatica* L.	1.49
Zhuling	*Polyporus umbellatus* (Pers.) Fr.	1.05
Tinglizi	*Draba nemorosa* L.	0.75
Fuling	*Poria cocos*(Schw) Wol	0.75

Trimetazidine (TMZ) (H20066534) was acquired from Reyoung Pharmaceutical Co. Ltd. (Shandong, China). Benzylpenicillin sodium for injection (H11302065) was obtained from North China Pharmaceutical Company, Ltd. (Hebei, China). Pyrrolidinedithiocarbamate ammonium (PDTC) (HY-18738) and resatorvid (TAK-242) (HY-11109) were obtained from MEDCHEMEXPRESS LLC (New Jersey, USA).

### Reagents

TLR4 Rabbit pAb (A5258) was purchased from ABclonal Biotechnology Co., Ltd. (Hubei, China);Anti-MyD88 antibody (ab219413), Anti-Glucose Transporter GLUT1 antibody (ab115730), Anti-Glucose Transporter GLUT4 antibody (ab33780) and Anti-MPC-1 antibody (ab74871) were purchased from Abcam (Cambridge, UK);NFKB1,p105,p50 Polyclonal antibody, ACC1 Polyclonal antibody, Phospho-ACC1 (Ser79) Polyclonal antibody, GAPDH Polyclonal antibody were purchased from Proteintech Group, Inc (Hubei, China);High Sensitivity Rat TNF-α (Tumor Necrosis Factor Alpha) ELISA Kit (E-HSEL-R0001), High Sensitivity Rat IL-1β (Interleukin 1 Beta) ELISA Kit (E-HSEL-R0002), High Sensitivity Rat IL-6 (Interleukin 6) ELISA Kit (E-HSEL-R0004), Rat NT-proBNP(N-Terminal Pro-Brain Natriuretic Peptide) ELISA Kit (E-EL-R3023), Rat BNP(Brain Natriuretic Peptide) ELISA Kit (E-EL-R0126), Rat ANP(Atrial Natriuretic Peptide) ELISA Kit (E-EL-R0017c), Rat LBP(Lipopolysaccharide Binding Protein) ELISA Kit (E-EL-R0589), Free Fatty Acids (NEFA/FFA) Fluorometric Assay Kit (E-BC-F039), L-Lactic Acid (LA) Colorimetric Assay Kit (E-BC-K044-M), Rat IL-18(Interleukin 18) ELISA Kit (E-EL-R0567c), Rat INS(Insulin) ELISA Kit (E-EL-R3034), Enhanced Cell Counting Kit 8 (CCK-8) (E-CK-A362)were purchased from Elabscience Biotechnology Co., Ltd. (Hubei, China);ATP Content Assay Kit (BC0305) was purchased from Beijing Solarbio Science & Technology Co.,Ltd. (Hubei, China);pentobarbital sodium (P3761), Lipopolysaccharides from Escherichia coli O111:B4 (L2630-10MG) was purchased from Merck KGaA (Darmstadt, Germany);Reactive Oxygen Species Assay Kit (S0033S)was purchased from Shanghai Biyuntian Biotechnology Co., Ltd. (Shanghai, China);Isoflurane (R510-22-10)was purchased from RWD Life science Co.,LTD. (Guangdong, China);2-NBDG, BODIPY 493/503 methyl bromide were purchased from MEDCHEMEXPRESS LLC (New Jersey, USA);

### Preparation and analysis of YXT

UHPLC-MS/MS analysis of YXT was conducted on a Vanquish UHPLC system (Thermo Fisher Scientific) fitted with a Waters UPLC BEH C18 column (1.7 μm, 2.1 × 100 mm). The injection volume was 5 μL, and the flow rate was maintained at 0.5 mL/min. The mobile phase consisted of 0.1% formic acid in water (A) and 0.1% formic acid in acetonitrile (B), with a linear gradient elution program as follows: 0–11 min, 85%–25% A; 11–12 min, 25%–2% A; 12–14 min, 2%–2% A; 14–14.1 min, 2%–85% A; 14.1–15 min, 85%–85% A; 15–16 min, 85%–85% A. An Exactive mass spectrometer coupled with Xcalibur software was utilized to acquire MS and MS/MS data using the information-dependent acquisition (IDA) mode. During each acquisition cycle, the mass range was set from 100 to 1500.

### Preparation of drug-containing serum and blank serum

We stratified 30 healthy male Sprague-Dawley rats weighing 200 ± 20 g into three groups: a blank serum group, a YXT drug-containing serum group, and a TMZ serum group. The YXT drug-containing serum group received a dosage of 5.6 g/kg·d, equivalent to three times the adult human dosage, for one consecutive week. The TMZ group received a dosage of 0.01 g/kg for one consecutive week. The blank serum group received distilled water through gavage daily for one week. Each rat received the respective treatment through gastric gavage 1 h before anesthesia. Anesthesia was induced using 2% sodium pentobarbital (0.26 mL/100 g). Using aseptic techniques, blood samples were collected from the abdominal aorta. After being left to stand for 2 hours, the samples underwent centrifugation at 3000 r/min for 15 minutes to isolate the serum. The resulting serum was then passed through a 0.22 µm microporous membrane, inactivated by heating at 56 °C for 30 minutes, and subsequently divided into aliquots, labeled, and preserved at –80 °C for subsequent analysis. The resulting extracts were subsequently analyzed via UHPLC-MS/MS under the chromatographic conditions detailed in section “Preparation and analysis of YXT.”

### Network pharmacological analysis

The absorbed bloodstream components of YXT identified using UHPLC-MS/MS were regarded as the primary active constituents (20). The corresponding targets were obtained from the ChEMBL database and the TCMIO database. Metabolites were evaluated according to their absorption rate and drug-likeness, with selection criteria of percent human oral absorption ≥ 30% and a drug-likeness value ≥ 0.18. Therapeutic targets for IHF were obtained from the TTD database and the GeneCards database. The targets of the absorbed components and the disease therapeutic targets were visualized using a Venn diagram.

The target proteins were imported into the STRING database, selecting “Homo sapiens” as the species and establishing a confidence score threshold > 0.9, to construct a protein-protein interaction (PPI) network. The node relationship data were imported into Cytoscape software (version 3.9) to generate a network graph, and core targets of YXT for IHF treatment were identified based on the DEGREE value. These core genes were subsequently uploaded to the gene ontology (GO) database and the Kyoto encyclopedia of genes and genomes (KEGG) pathway database for enrichment analysis, aimed at clarifying the pathways through which the active components of YXT exert their therapeutic effects on IHF.

### Molecular docking

Molecular docking is essential in identifying active ingredients and their corresponding targets in TCM. Molecular docking simulates the interactions between TCM molecules and target proteins, allowing for the prediction and evaluation of the binding affinity and modes of TCM components with potential targets. This approach aids in elucidating the mechanisms of action of TCM and facilitates the identification of highly active and specific compounds, thus offering a scientific basis for further drug development and optimization. This analysis utilized a Galaxy workflow that incorporates the molecular docking platform AutoDock Vina (Hands-on: Protein-ligand docking/computational chemistry). Protein PDB structure files were obtained from protein databases, including AlphaFold (https://alphafold.ebi.ac.uk/), while the 3D SDF structures of ligands were downloaded from the PubChem compound database. Proteins were preprocessed by adding hydrogen atoms, and a ligand compound library was created by identifying structurally similar compounds in the ChEMBL database. Binding pockets in the protein structures were identified using RDKit, followed by docking each compound in the library into the protein to determine the optimal binding pose and binding energy.

### Animal model

A total of 120 healthy adult male SPF-grade Sprague-Dawley rats weighing 200–220 g were utilized to create a post-myocardial infarction HF model by ligating the left anterior descending coronary artery, following the methodology established in our previous research (19). Anesthesia was induced after body weight measurement through an intraperitoneal injection of 2% sodium pentobarbital (0.26 mL/100 g). Tracheal intubation was conducted, and the patient was connected to a ventilator with a respiratory rate of 80 breaths/min and a tidal volume of 7–9 mL. Thoracotomy was performed, and a needle was inserted at the marginal branch of the coronary artery adjacent to the left atrial appendage, exiting 1 mm inferior to the pulmonary artery cone. A 6/0 suture was used to ligate the coronary artery and the adjacent vein, followed by immediate closure of the chest. In the sham group, a small loop was created at the same anatomical location without ligation. Electrocardiography confirmed successful ligation by detecting ST-segment elevation in the precordial leads. Postoperatively, each rat received a daily intramuscular injection of 400,000 units of penicillin for three consecutive days to prevent infection. After model establishment, animals were routinely maintained for four weeks. Echocardiography was performed at the end of the fourth week, and a left ventricular ejection fraction (LVEF) of < 45% was utilized as the criterion for successful model induction.

### Animal grouping and drug administration

Rats that met the modeling criteria were randomly assigned using a random number table to one of the following groups: model, low-dose YXT (1.4 g/kg), medium-dose YXT (2.8 g/kg), high-dose YXT (5.6 g/kg), and TMZ (0.01 g/kg). All drugs were dissolved in normal saline and administered via gavage. The sham and model groups received an equal volume of normal saline. The gavage volume for all groups was standardized at 10 mL/kg daily for four consecutive weeks. After treatment, the rats were fasted for 12 h with free access to water. Anesthesia was induced using 2% pentobarbital sodium. Blood samples were collected from the abdominal aorta and allowed to stand for 1 h. Subsequently, the samples were centrifuged at 3500 r/min for 10 min at 4 °C. The supernatant was collected and stored at –80 °C. Hearts were excised and preserved in 4% paraformaldehyde or at –80 °C for further analysis.

### Echocardiographic analysis

A small animal color Doppler ultrasound system was employed to assess cardiac function following four weeks of treatment. Anesthesia was initiated with 3% isoflurane and subsequently maintained at 1%. While the rats were positioned supine, echocardiographic measurements were taken, including left ventricular end-systolic diameter (LVIDs), left ventricular end-diastolic diameter (LVIDd), LVEF, and left ventricular fractional shortening (FS).

### Histological and morphological analysis

Heart specimens were immersed in 4% paraformaldehyde for 48 hours, then dehydrated through a graded series of ethanol and xylene, and finally embedded in paraffin. Serial sections of 4–5 μm in thickness were prepared and stained with hematoxylin and eosin (H&E) as well as Masson’s trichrome, followed by image capture under a light microscope.

### Transmission electron microscope

Myocardial tissue specimens, with a volume typically not exceeding 1×1×1 mm, were rapidly immersed in electron microscopy fixative and fixed at 4 °C for 2–4 h. Subsequently, the samples were subjected to post-fixation, dehydration, infiltration, embedding, sectioning, and staining. The ultrastructure of rat cardiac tissue was observed using a transmission electron microscope (HT7700, Hitachi), and images were acquired for subsequent analysis.

### Central carbon metabolism analysis

Central carbon metabolism analysis was performed according to a previously described protocol (21). In brief, myocardial samples were mixed with 500 mL of extract solution (MeOH/H_2_O, 3:1, v/v, precooled at –40 °C), sonicated in an ice-water bath for 10 minutes, and then incubated at –40 °C for 1 hour, followed by centrifugation at 12,000 rpm and 4 °C for 15 minutes. The resulting supernatant was collected for HPIC-MS/MS analysis.

The HPIC separation was performed utilizing a Thermo Scientific Dionex ICS-6000 HPIC System (Thermo Scientific), equipped with Dionex IonPac AS11-HC (2 × 250 mm) and AG11-HC (2 × 50 mm) columns. The mobile phase A was 100 mM NaOH in water, and the mobile phase D was ultrapure water. A separate pumping system was employed to supply the solvent (2 mM acetic acid in methanol), which was mixed with column effluent before entering ESI (flow rate of 0.15 mL/min). The column temperature was set at 30 °C, and the auto-sampler temperature was set at 4 °C. The injection volume was 5 μL. An AB SCIEX 6500 QTRAP+ triple quadrupole mass spectrometer (AB Sciex) equipped with an ESI interface was applied for assay development.

### Quantitative lipidomic analysis

Quantitative lipidomic analysis was performed according to a previously described protocol (22). In brief, approximately 20 mg of each sample was weighed into an Eppendorf tube on dry ice. After adding myocardial samples were mixed with 480 mL of extraction solvent (methyl tert-butyl ether [MTBE]: MeOH = 5:1)containing internal standard, sonicated in an ice-water bath for 10 minutes, and then incubated at –40 °C for 1 hour, followed by centrifugation at 3000 rpm and 4 °C for 15 minutes. The resulting supernatant was collected for HPIC-MS/MS analysis.

The UHPLC separation was performed using an ACQUITY Premier Series UHPLC system. The mobile phase A comprised 40% water, 60% acetonitrile, and 10 mmol/L ammonium formate. The mobile phase B comprised 10% acetonitrile, 90% isopropanol, and 10 mmol/L ammonium formate. The column temperature was maintained at 45 °C. The auto-sampler temperature was 10 °C, and the injection volume was 2 μL. A SCIEX Triple Quad™ 6500+ mass spectrometer was utilized for assay development. SCIEX Analyst Work Station Software (Version 1.6.3) and DATA DRIVEN FLOW (Version 1.0.1) were utilized for the target compounds quantification.

### Assessment of glucose metabolism by positron emission tomography/computed tomography imaging

Myocardial fluorodeoxyglucose (FDG) imaging of different layers in living rats was conducted using small animal PET/CT to quantitatively assess the metabolic rate and analyze the metabolic characteristics of rat myocardial tissue. Before scanning, rats in each group were fasted for over 12 h to enhance tracer uptake in the myocardium. Subsequently, 18F-FDG (80 MBq/kg) was administered through tail vein injection. Rats were anesthetized with 1%–1.5% isoflurane, and positron emission tomography–computed tomography (PET-CT) imaging was performed using the Mediso nanoScan SPECT/CT system. PET-CT images were obtained 40 min post-injection. The standardized uptake value (SUV) was analyzed using Inveon Research Workplace software, calculated as SUV = C/(D/M), where C represents the activity concentration in the region of interest (ROI), D denotes the injected dose, and M indicates body weight. The mean, minimum, and maximum SUV values (SUVmean, SUVmin, SUVmax) were obtained.

### Cell culture and treatment

H9c2 cardiomyocytes were obtained from Procell Life Science & Technology Co., Ltd. (Wuhan, China; Catalog No: CL-0089). The cryopreserved H9c2 cells were obtained from a –80 °C freezer and quickly thawed in a 37 °C water bath. The cells were subsequently transferred to a sterile biological safety cabinet, combined with complete culture medium, and centrifuged to yield a cell pellet. The pellet was resuspended in complete medium and transferred to a culture flask, which was placed in a cell culture incubator. Cell morphology and density were monitored microscopically. Upon reaching 80%–90% confluence, cells were subcultured using trypsin.H9c2 cells were divided into the following experimental groups: control, model, YXT, TMZ, TAK-242, PDTC, YXT + TAK-242, and YXT + PDTC groups.

### Cell viability

H9c2 cell viability was determined using the Cell Counting Kit-8 (CCK-8, Elabscience, E-CK-A362). LPS was prepared as a stock solution in DMEM and stored at –80 °C. Cells in logarithmic growth phase were seeded into 96-well plates at a density of 5 × 10^4^ cells/mL. The LPS stock was vortexed and then diluted to final concentrations of 1, 5, 10, 25, 50, and 100 μg/mL. These dilutions were added to H9c2 cells, followed by co-incubation in a cell culture incubator (5% CO_2_, 37 °C) for 12, 24, or 48 h. Subsequently, 10 μL of CCK-8 solution was added to each well, and the plates were incubated in the dark for 1 h. Absorbance at 450 nm was measured using a microplate reader, and cell viability was calculated accordingly to identify the optimal LPS concentration and exposure time for model establishment.

Based on the above findings, the LPS-induced model was established. Cells were then treated with blank serum, YQX-containing serum, or TMZ-containing serum at concentrations of 0%, 5%, 10%, 15%, and 20%, and co-incubated under the same conditions (5% CO_2_, 37 °C) for 12, 24, or 48 h. The TLR4 inhibitor TAK-242 and the NF-κB inhibitor PDTC were dissolved and diluted to graded concentrations (5, 25, 50, 100, 500, and 1,000 μM), added to H9c2 cardiomyocytes, and incubated at 37 °C for 24 h. Cell viability was again assessed using the CCK-8 kit to determine the optimal concentrations of TAK-242 and PDTC, as well as the optimal concentration and intervention time for the drug-containing sera.

### Flow cytometric assays

Cellular uptake of 2-NBDG and BODIPY 493/503, as well as intracellular ROS levels, were measured by flow cytometry(Beckman, A00-1-1102).

Probe preparation: For 2-NBDG, a 1 mM stock was prepared by dissolving 1 mg in 2.92 mL DDH_2_O. For BODIPY 493/503, a 10 mM stock was obtained by dissolving 1 mg of its methyl bromide salt in 382 μL anhydrous DMSO. For ROS detection, a 10 mM DCFH-DA stock was used. All stocks were diluted with basal medium (or serum-free culture medium for DCFH-DA) to a final working concentration of 10 μM.

Cell staining and analysis: After trypsinization, cells from each group were collected and washed twice with PBS (centrifugation at 1500 rpm for 5 min each wash). The cells were then incubated with the appropriate working solution as follows: 2-NBDG at room temperature for 30 min; BODIPY 493/503 at room temperature for 20 min; DCFH-DA at 37 °C for 30 min. Following incubation, the cells were again washed twice with PBS under the same centrifugation conditions. Finally, the stained cells were analyzed using a flow cytometry.

### Measurement of biochemical indices

Fresh myocardial tissue was homogenized in an ice bath and centrifuged at 10,000 × g and 4 °C for 3 min, with the supernatant collected for subsequent analysis. H9c2 cells were washed with PBS, disrupted through ultrasonication, and centrifuged at 10,000 × g and 4 °C for 10 min, after which the supernatant was collected. Fresh whole blood from rats was allowed to stand at room temperature for 2 h, followed by centrifugation at 3,000 rpm and 2–8 °C for 15 min to obtain serum. Fasting blood glucose (FBG) and glycated serum protein (GSP) serum samples were assessed using an automatic biochemistry analyzer (Chemray 240, Rayto, China). Total cholesterol (TC), triglyceride (TG), high-density lipoprotein cholesterol (HDL-C), and low-density lipoprotein cholesterol (LDL-C) in serum were measured using an automatic biochemistry analyzer. Creatinine kinase MB isoenzyme (CK-MB) and lactate dehydrogenase 1 (LDH1) levels in rat serum and H9c2 cell supernatant were measured with an automatic biochemistry analyzer. The free FA (FFA) levels in rat serum and H9c2 cell supernatant were detected using fluorometry according to the kit instructions, lactate levels were determined using colorimetry, and ATP content in myocardial tissue and H9c2 cells was measured using a micro assay method.

### Enzyme-linked immunosorbent assay

Serum levels of fasting insulin (Fins), LPS, atrial natriuretic peptide(ANP), B-type natriuretic peptide (BNP), and N-terminal pro-B-type natriuretic peptide (NT-proBNP) in rats were measured using the ELISA. Additionally, the concentrations of TNF-α, IL-1β, IL-6, and IL-18 in rat serum and cell culture supernatant were assessed. The quantitative insulin sensitivity check index (QUICKI) and homeostasis model assessment of insulin resistance (HOMA-IR) were calculated using the following formulas: QUICKI = 1/(log FBG + log Fins), HOMA-IR = (FBG × Fins)/22.5.

### Reverse transcription quantitative polymerase chain reaction

Total RNA was extracted from mouse cardiac tissues and H9C2 cells utilizing TRIzol reagent (G3013, Servicebio, China). The total RNA concentration and purity were assessed using a nucleic acid/protein analyzer. Subsequently, reverse transcription was conducted by assembling the reaction system according to the manufacturer’s instructions for the reverse transcription kit, and the reaction was executed using a PCR instrument. Quantitative real-time PCR was performed following the protocol utilizing the SYBR Green qPCR kit (G3337, Servicebio, China) on a CFX Connect Real-Time PCR System (Bio-Rad, USA). GAPDH served as the reference housekeeping gene, and the expression levels of each target gene were normalized to GAPDH levels. The relative expression of target genes to GAPDH was calculated using the 2^−ΔΔCT^ method. **Table 2** presents the primer sequences.

**Table 2:** Primer sequences for real-time PCR.

Target gene	Primer sequence(5’to3’)
R-GAPDH-S	CTGGAGAAACCTGCCAAGTATG
R-GAPDH-A	GGTGGAAGAATGGGAGTTGCT
R-TLR4-S	CCAGGTGTGAAATTGAGACAATTG
R-TLR4-A	AAGCTGTCCAATATGGAAACCC
R-myd88-S	TCGACGCCTTCATCTGCTAC
R-myd88-A	CCATGCGACGACACCTTTTC
R-NFKB1 (5)-S	GACAGCAGGACCCAAGGACA
R-NFKB1 (5)-A	CTGTAGATAGGCAAGGTCAGAATG

### Western blot analysis

Cardiac tissues were homogenized in radio immuno precipitation assay lysis buffer (G2002-100ML, Servicebio, China) with an electric homogenizer; cells were scraped from the growth surface and collected in the lysis buffer. After incubation on ice for 15 min, the samples were centrifuged at 16,000 × g for 15 min at 4 °C to obtain the cytosolic protein fraction. Protein concentrations in the non-denatured lysates were determined using a bicinchoninic acid assay kit. Aliquots of 20 μg of protein were separated on 12% SDS-polyacrylamide gels and then transferred onto PVDF membranes. Following blocking with 5% skim milk for 2 h and washing with Tris-buffered saline containing Tween 20 (TBST), the membranes were incubated overnight at 4 °C with primary antibodies against GAPDH (1:3000), TLR4 (1:3000), MyD88 (1:3000), NF-κB P50 (1:3000), p-NF-κB P50 (1:3000), GLUT1 (1:3000), GLUT4 (1:3000), MPC-1 (1:3000), ACC1 (1:3000), and p-ACC1 (1:3000). The following day, the membrane was washed thrice with TBST and subsequently incubated with an HRP-conjugated secondary antibody (GB23303, Servicebio, China) at 37 °C for 1 h. Protein expression was visualized using an ultrasensitive enhanced chemiluminescence substrate, and data were analyzed with the AIWBWellTM analysis software (Servicebio, China).

### Statistical analysis

Statistical analysis of all relevant data was conducted using IBM SPSS Statistics (Version 23). Graphical representations were created with GraphPad Prism (version 7). Quantitative data are presented as mean ± standard deviation. Intergroup differences were evaluated using one-way analysis of variance, followed by the LSD test for equal variances or Dunnett’s T3 test for unequal variances. A significance threshold of *P* < 0.05 was applied.

## Results

### Content of active ingredients in YXT

We extracted 2,581 active ingredients from the YXT, comprising 353 prenol lipids, 212 terpenoids, 135 steroids and steroid derivatives, 113 flavonoids, 107 fatty acyls, 103 organooxygen compounds, 92 phenylpropanoids, 67 triterpenoids, and 64 alkaloid. Meanwhile, we extracted 1,055 active ingredients from the YXT-containing serum, comprising 45 terpenoids, 51 flavonoids, 26 phenylpropanoids, 10 alkaloids, and 10 phenolic compounds. Additionally, 20 components were identified with high confidence (**Figure 1**, **Table 3**).

**Figure 1:**
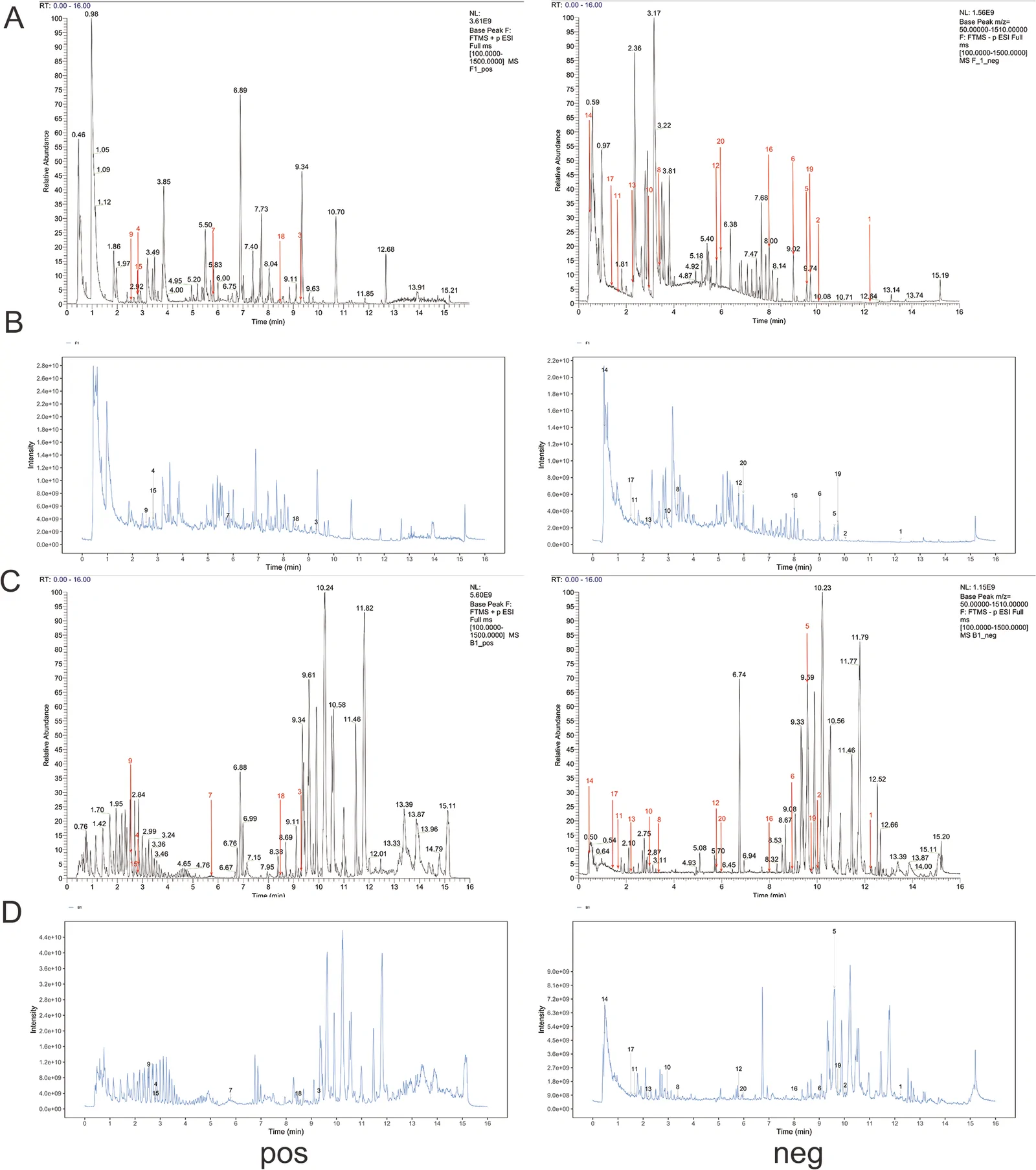
Identification of chemical components of YXT by UHPLC-MS/MS. **(A, B)** Base peak chromatogram **(A)** and total ion chromatogramc **(B)** of YXT in positive and negative ion modes. **(C, D)** Base peak chromatogram **(C)** and total ion chromatogramc **(D)** of YXT-containing serum in positive and negative ion modes.

**Table 3:** Identification information of chemical components of YXT.

No.	NameEN	Formula	RTmin	Mode	MS2	mzmed	ppm	Class	Level
1	Pachymic acid	C_33_H_52_O_5_	733.7295	neg	527.372075;528.372772;58.599905;465.335631;514.926227	527.374154142626	0.292283239121722	Terpenoids	I
2	Pygenic acid A b	C_30_H_48_O_4_	601.845	neg	471.343419;472.353646;52.374424;409.312103;91.968282	471.34812592276	0.26715447259955	Terpenoids	I
3	Cryptotanshinone	C_19_H_20_O_3_	557.9945	pos	297.149913;298.153235;251.144301;279.137647;254.092007	297.148300480055	1.01121242996645	Diterpenoids	II
4	Guanosine	C_10_H_13_N_5_O_5_	169.322	pos	284.100029;285.074497;91.973148;83.021578;270.05043	284.100072673317	0.255801824719149	Diterpenoids	II
5	Linoelaidic acid	C_18_H_32_O_2_	575.865	neg	279.233361;280.236046;91.966734;59.013959;261.221054	279.232772434914	0.814965535773232	Aliphatic acyl	II
6	Alisol A	C_30_H_50_O_5_	540.952	neg	535.36516;536.366286;471.353712;489.354231;59.48808	535.364160725734	0.300217582262109	Triterpenoids	I
7	Boldine	C_19_H_21_NO_4_	347.764	pos	350.13575;351.213177;349.199344;259.204408;333.202085	350.136186031083	0.531310645433381	Alkaloid	I
8	Formononetin	C_16_H_12_O_4_	202.63	neg	267.067893;252.043573;268.068607;223.039315;251.033227	267.066477713054	1.78874210683917	Isoflavones	I
9	Isorhamnetin	C_16_H_12_O_7_	152.402	pos	317.06577;302.043847;318.069412;303.047333;274.04658	317.064934649749	0.206109991609703	Flavonoids	II
10	Salicylic acid	C_7_H_6_O_3_	177.556	neg	93.034816;137.024227;138.019721;94.037737;108.021758	137.024237715652	1.73484382000041	Phenols	II
11	Trans-4-Coumaric acid	C_9_H_8_O_3_	99.59845	neg	119.049629;163.040379;120.053838;164.044298;147.044919	163.039765787216	1.43653778489536	phenylpropanoids	II
12	Astragaloside IV	C_41_H_68_O_14_	347.675	neg	829.459263;829.504582;830.503354;91.96923;71.013932	829.458325296812	2.01903234558234	Terpenoids	I
13	Gluconapin	C_11_H_19_NO_9_S_2_	131.975	neg	96.959561;372.041187;74.990673;95.952323;79.957447	372.042376532355	1.67579739393857	Carbohydrates and derivatives	I
14	Sorbitol	C_6_H_14_O_6_	28.0768	neg	181.07206;71.013957;101.024038;59.014019;89.024464	181.071572155007	2.36285015867735	ChemicalBook	II
15	CAFFEATE	C9H8O4	168.82	pos	163.039605;135.043562;145.029047;117.033639;89.038296	163.038945046238	0.337059111607628	Phenylpropanoids	II
16	Ginsenoside Rg3	C_42_H_72_O_13_	480.099	neg	783.494416;71.013896;101.023965;783.515218;113.024701	783.489495609528	0.643774389829884	Terpenoids	I
17	Quercetin-3-O-glucoside	C_21_H_20_O_12_	90.6527	neg	463.08774;301.034209;300.026099;271.023392;272.033611	463.089188278632	0.40657099484654	Flavonoids	I
18	Tanshinone IIb	C_19_H_18_O_4_	508.9575	pos	311.126285;267.136055;185.0959;312.133329;225.089975	311.127858460714	0.45492321615175	Naphthalene Quinones	I
19	Ginsenoside Rg5	C_42_H_70_O_12_	583.9125	neg	765.484191;71.013465;101.02413;765.50428;113.023781	765.478379123848	0.811095608989611	Terpenoids	I
20	Ginsenoside Re	C_48_H_82_O_18_	358.604	neg	945.535907;101.024073;71.013953;113.02491;89.024381	945.542939446522	0.0640409604249506	Terpenoids	I

### Network pharmacology analysis

We analyzed the active ingredients identified in the YXT-containing serum to identify the potential targets of YXT using network pharmacology analysis. Metabolite targets were retrieved from ChEMBL and TCMIO databases, and metabolites were evaluated based on absorption rate and drug-likeness, resulting in 312 targets for YXT active ingredients. Genes associated with HF were identified using GeneCards and TTD databases. After integration and elimination of duplicates, 2019 disease-related genes were identified. The intersection of drug targets from YXT and HF targets yielded 200 overlapping target genes (**Figure 2A**).

**Figure 2:**
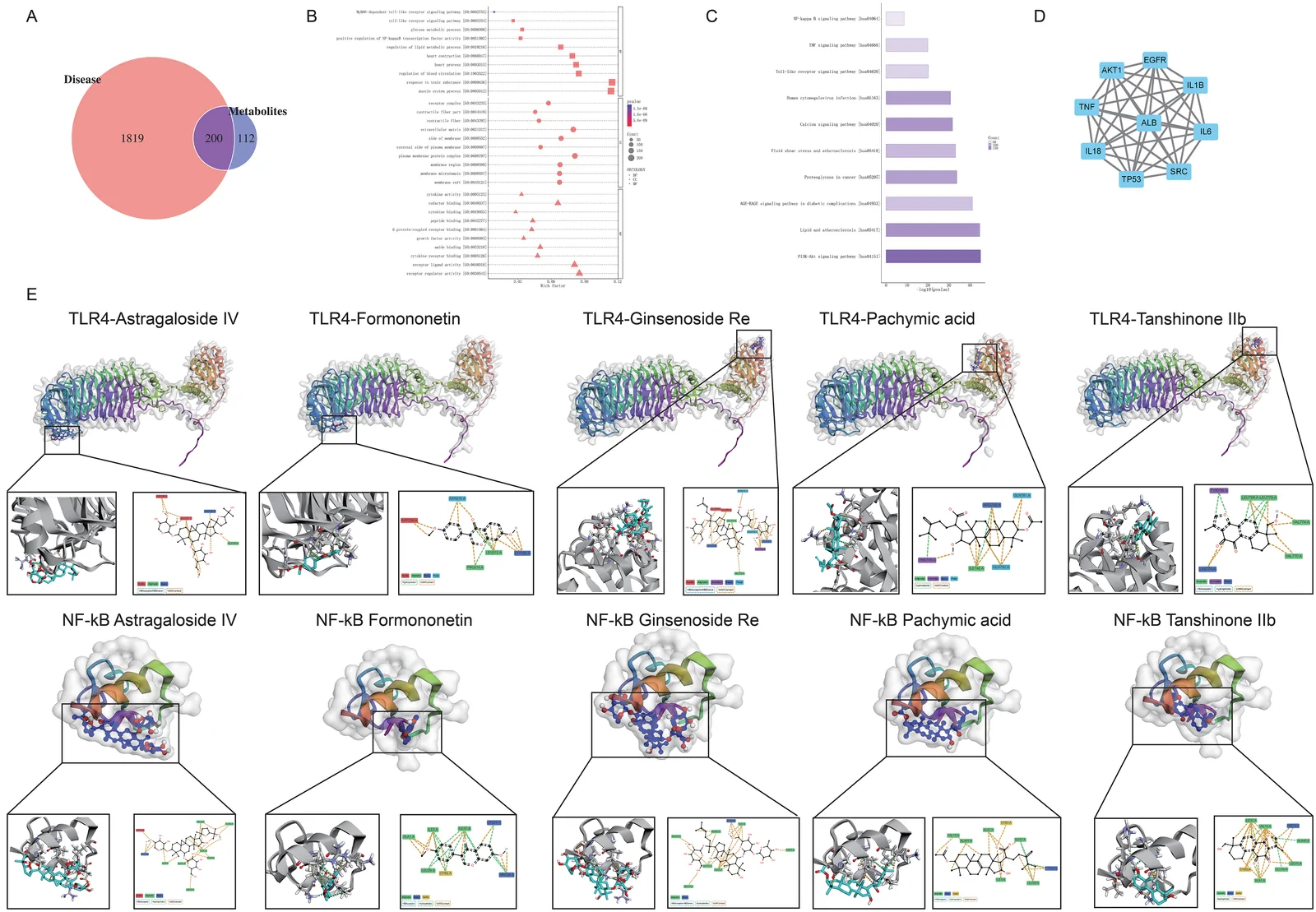
Network pharmacology predicts the effect of YXT on HF and molecular docking results. **(A)** Venn diagram of the targets of YXT and HF **(B, C)** GO and KEGG enrichment analyses of drug-disease targets. **(D)** PPI network of drug-disease interactions. **(E)** Molecular docking results.

Enrichment analysis was conducted on the 200 targets using a screening criterion of *P* < 0.05, leading to the identification of 6,970 GO functional terms and 246 KEGG pathways. The results of GO enrichment analysis are illustrated in the **Figure 2B**, highlighting pathways, including the MyD88-dependent toll-like receptor signaling pathway, toll-like receptor signaling pathway, glucose metabolic process, positive regulation of NF-kappaB transcription factor activity, and regulation of lipid metabolic process. The KEGG enrichment analysis results are illustrated in the **Figure 2C**, highlighting the NF-kappa B signaling pathway, toll-like receptor signaling pathway, and TNF signaling pathway. Subsequently, the STRING database was employed to construct a protein–protein interaction network for the target proteins, which included IL18, IL6, IL1B, and TNF (**Figure 2D**). Previous research findings from our group and literature indicate that the TLR4/NF-κB pathway regulates inflammatory factors; thus, the TLR4/NF-κB pathway was selected for investigation. Molecular docking was performed between the key targets TLR4 and NF-κB and the five active compounds, namely astragaloside IV, Ginsenoside_Re, pachymic acid, formononetin, and tanshinone IIb (**Table 4**). Docking score > |4.25| indicates average binding activity, > |5.0| suggests good binding activity, and > |7.0| reflects strong binding activity (23). All five active compounds demonstrated favorable binding affinity to proteins encoded by TLR4 and NF-κB. Notably, Ginsenoside_Re exhibited the most favorable binding energy, indicating particularly strong interactions with both targets (**Figure 2E**).

**Table 4:** Binding energy table of key components and key targets.

Key components	Key targets	Binding energy
Astragaloside_IV	TLR4	-8.814 kcal/mol
_Formononetin	TLR4	-6.832 kcal/mol
Ginsenoside_Re	TLR4	-7.32 kcal/mol
Pachymic_acid	TLR4	-9.128 kcal/mol
Tanshinone_IIb	TLR4	-7.336 kcal/mol
Astragaloside_IV	NF-κB	-6.298 kcal/mol
_Formononetin	NF-κB	-5.571 kcal/mol
Ginsenoside_Re	NF-κB	-6.5 kcal/mol
Pachymic_acid	NF-κB	-5.798 kcal/mol
Tanshinone_IIb	NF-κB	-5.021 kcal/mol

### Effect of YXT on cardiac function in HF rat model

To evaluate whether YXT improves cardiac function in a rat model of HF, echocardiography was performed. As shown in **Figure 3A**, compared with the sham group, the model group showed markedly decreased EF and FS values (*P* < 0.01), along with markedly increased LVIDs and LVIDd (P < 0.01). Relative to the model group, all intervention groups exhibited significantly elevated EF and FS values (*P* < 0.01). LVIDd was reduced in the low-dose YXT group (*P* < 0.05), whereas the medium-dose YXT, high-dose YXT, and TMZ groups showed a more pronounced reduction (*P* < 0.01). Furthermore, LVIDs decreased significantly in all intervention groups compared with the model group (*P* < 0.01).

**Figure 3:**
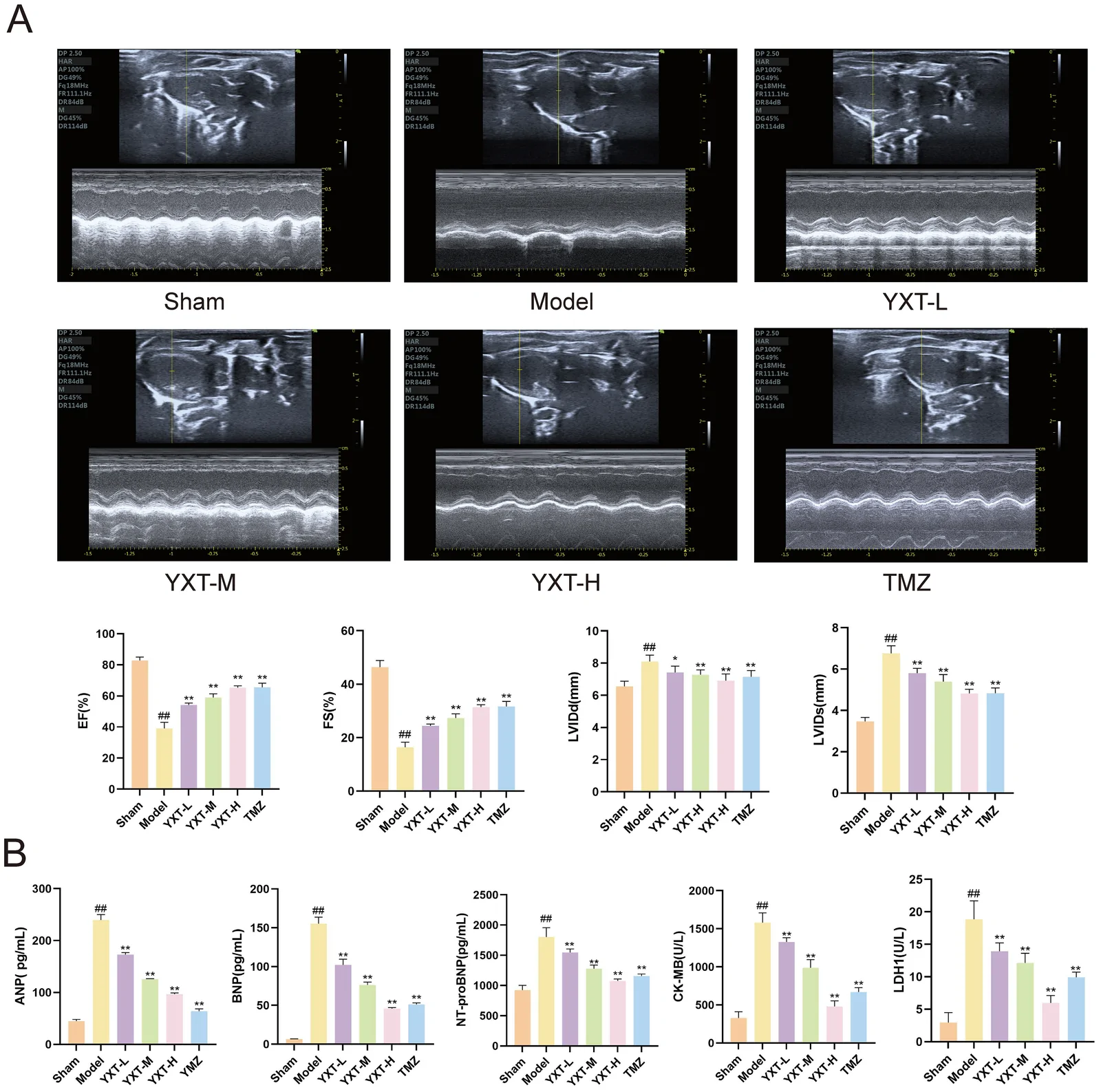
YXT improves cardiac function and serum enzymes indicators. **(A)** Echocardiography measurements of LVEF, LVFS, LVEDD, LVIDd, LVIDs (n = 6). **(B)** Serum enzymes indicators (n = 6). ^##^*P<*0.01 vs. sham group; **P<0.01 vs. model group.

Serum levels of NTproBNP, BNP, and ANP—key biomarkers of HF—were also assessed (**Figure 3B**). The model group had significantly higher levels of these biomarkers than the sham group (*P* < 0.01), while all intervention groups showed marked reductions relative to the model group (*P* < 0.01). Together with the echocardiographic findings, these results suggest that YXT enhances cardiac function in HF rats.

To determine whether YXT alleviates myocardial injury, serum biomarkers of cardiac damage (CK-MB and LDH1) were measured using biochemical assays. As illustrated in **Figure 3B**, the model group exhibited significantly elevated CK-MB and LDH1 levels compared with the sham group (*P* < 0.01), whereas all intervention groups showed significant reductions in both markers relative to the model group (*P* < 0.01). These data indicate that YXT attenuates myocardial injury in the HF rat model.

### Effect of YXT on myocardial pathological remodeling and mitochondrial structure in HF rat model

To investigate whether YXT improves cardiomyocyte morphology and the extent of myocardial fibrosis in HF rat model, H&E staining was performed to observe cardiomyocyte morphology, and Masson staining was utilized to assess the degree of myocardial fibrosis across groups. In H&E staining, nuclei appeared blue, whereas the cytoplasm appeared red. In Masson staining, collagen fibers were stained blue, whereas muscle fibers, fibrin, and red blood cells appeared red.

H&E staining revealed that the sham group exhibited normal tissue architecture devoid of degeneration or necrosis, which indicated no significant inflammatory cell infiltration in the interstitial space. The model group exhibited disorganized fiber arrangement, pyknosis, or even fragmentation and loss of cardiomyocyte nuclei, enlarged interstitial spaces, proliferation of fibrous connective tissue, and inflammatory cell infiltration compared to the sham group. Low- and medium-dose YXT groups exhibited more orderly fiber arrangement, with only occasional nuclear pyknosis or fragmentation, and persistent inflammatory cell infiltration and fibrous connective tissue proliferation than the model group. In high-dose YXT and TMZ groups, myocardial fibers were largely well-arranged, with only minor lymphocyte infiltration, no substantial nuclear pyknosis, and no cardiomyocyte fragmentation (**Figure 4A**).

**Figure 4:**
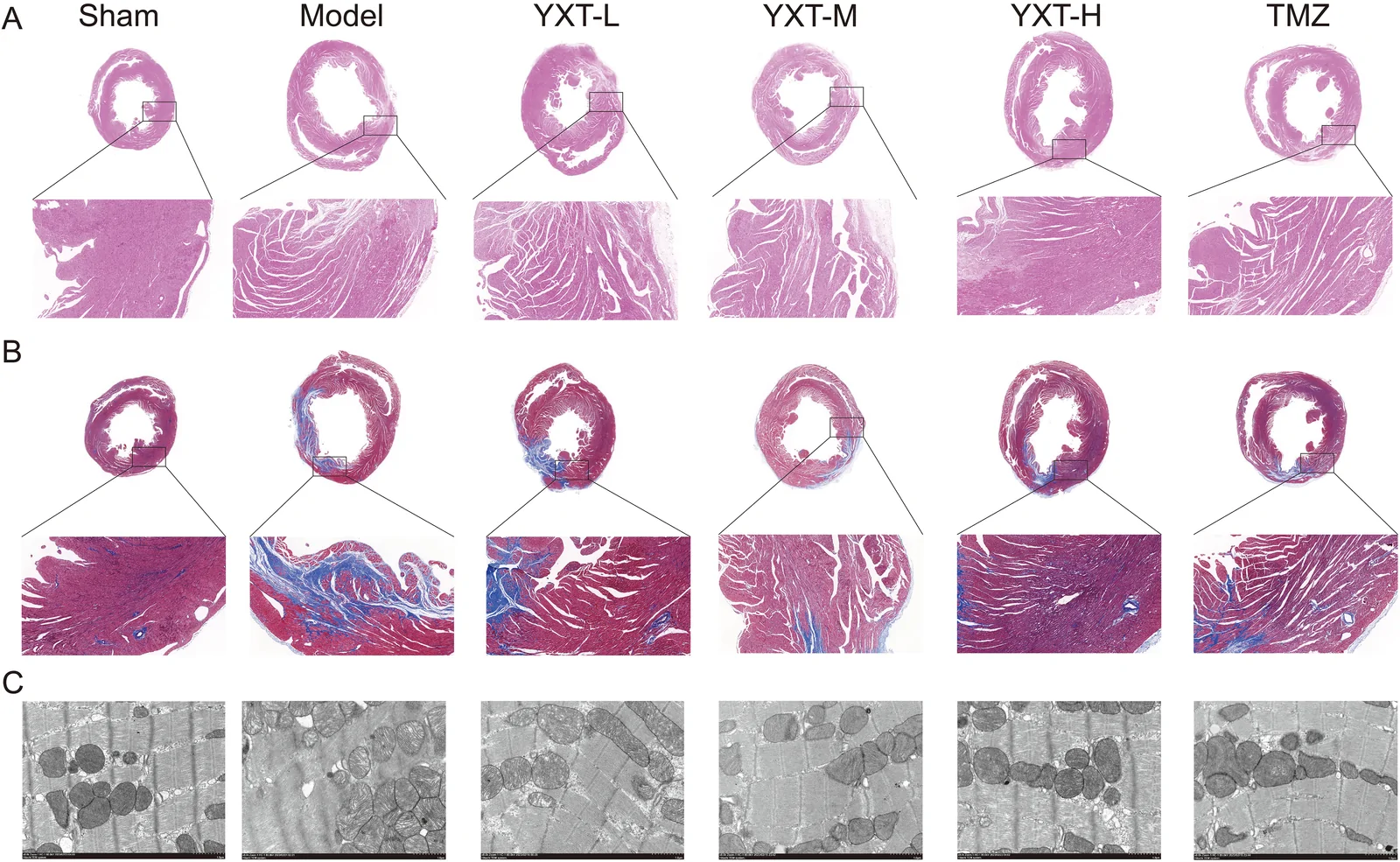
Myocardial pathological remodeling and mitochondrial structure were inhibited by YXT in HF rats. **(A)** HE staining(×100,bar=100μm); **(B)** Masson staining (×100,bar=100μm); **(C)** Morphological structure diagram of mitochondria (×8000,bar=1μm).

Masson staining revealed that, aside from minimal fibrous tissue proliferation surrounding large cardiac vessels and normal endocardium/epicardium in the sham group, no substantial fibrosis was observed. The model group exhibited a markedly increased blue-stained collagen fiber region in cardiac tissue compared to the sham group. Low-, medium-, and high-dose YXT groups exhibited dose-dependent progressively reduced blue-stained collagen fiber regions compared to the model group. High-dose YXT and TMZ groups exhibited comparable collagen fiber areas (**Figure 4B**).

Transmission electron microscopy was utilized to examine mitochondrial ultrastructure in the failing myocardium of rats. In the sham group, myocardial mitochondria exhibited regular arrangement and homogeneous size, without swelling or deformation, with clear and intact cristae and well-organized sarcomeres and overall structure. The model group exhibited inflamed and hypertrophied mitochondria of diverse morphologies, disrupted and dissolved cristae, and disorganized myofibrils. In low- and medium-dose YXT groups, partial mitochondrial edema and heterogeneous morphology were observed, although less severe than in the model group; cristae exhibited mild disorganization, and sarcomeres were relatively orderly. In high-dose YXT and TMZ groups, mitochondria were relatively well-arranged, with mild edema and deformation, partially unclear cristae, and relatively organized sarcomeres; overall morphological changes were milder than in the other treatment groups (**Figure 4C**).

YXT alleviates myocardial injury and attenuates myocardial fibrosis in HF rat model.

### Effects of YXT on LPS/TLR4/NF-κB pathway and inflammatory cytokines in HF rat model

To examine the effect of YXT on the LPS/TLR4/NF-κB pathway in an HF rat model, the expression levels were evaluated using RT-qPCR and Western blot analysis. The model group exhibited significantly elevated serum LPS levels and increased mRNA expression of TLR4, Myd88, and NF-κB p50 in myocardial tissue compared to the sham group (*P <* 0.01; **Figures 5A, B**). All intervention groups exhibited a significant reduction in the mRNA expression of TLR4, Myd88, and NF-κB p50 in myocardial tissue compared to the model group (*P <* 0.01; **Figures 5A, B**). Moreover, protein expression levels of TLR4, Myd88, NF-κB p50, and phosphorylated NF-κB p50 (p-NF-κB p50) were significantly increased in the model group than in the sham group (*P <* 0.01; **Figures 5A, B**). However, these protein expression levels were reduced in all intervention groups compared with the model group (*P <* 0.01; **Figures 5A, B**). These results indicate that the LPS/TLR4/NF-κB pathway is activated in HF rat model, and YXT can inhibit this pathway.

**Figure 5:**
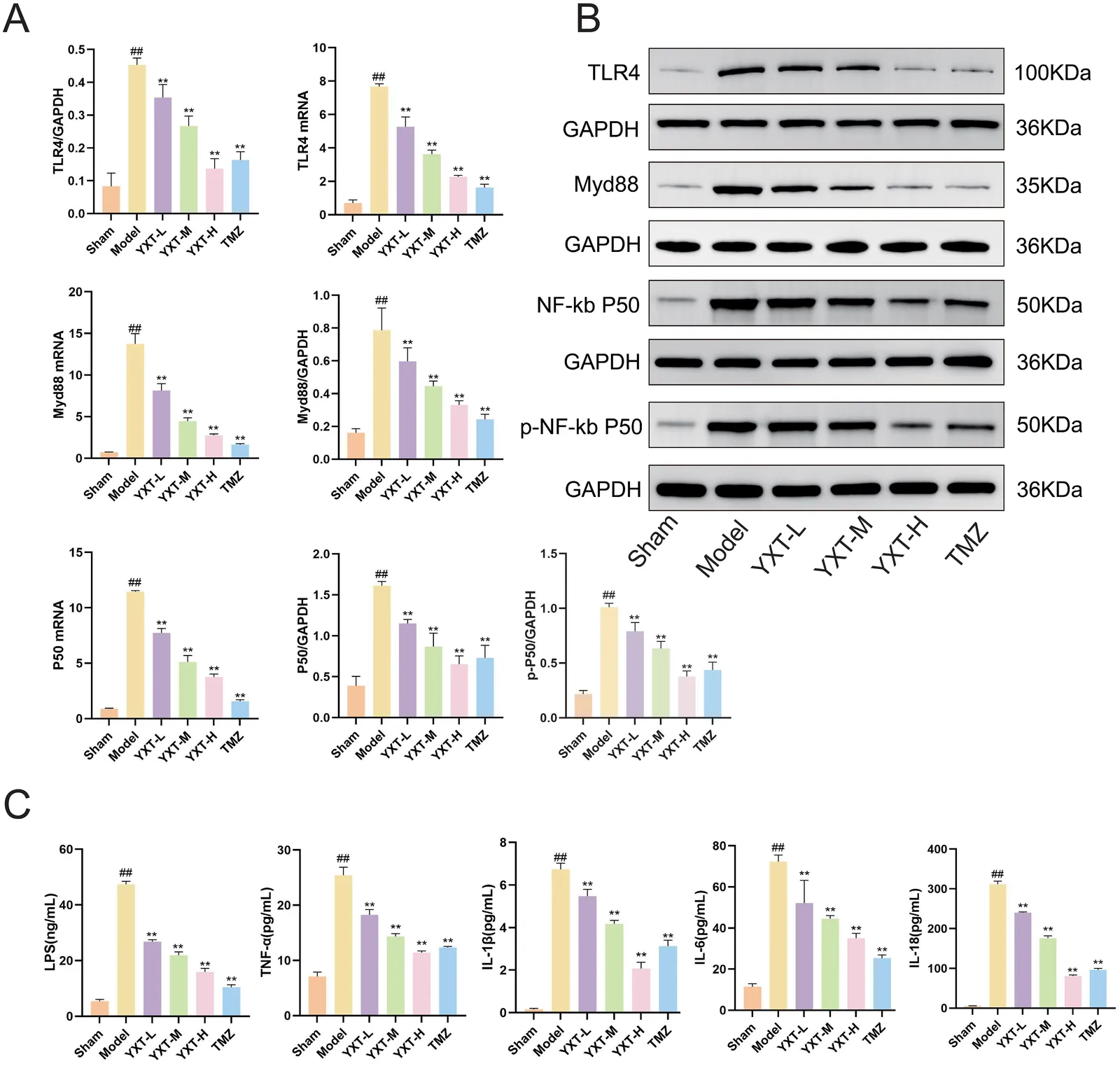
Effects of YXT treatment on the expression levels of the LPS/TLR4/NF-κB pathway in HF rat model. **(A, B)** Expression levels of LPS/TLR4/NF-κB pathway gene and protein(n=3). **(C)** Expression levels of LPS and inflammatory cytokines(n=6). ^##^*P<*0.01 vs. sham group; **P<0.01 vs. model group.

To determine whether YXT ameliorates inflammation in HF rat model, serum levels of inflammatory cytokines, including IL-1β, IL-6, IL-18, and TNF-α, were measured using ELISA. The model group exhibited significantly higher serum levels of IL-1β, IL-6, IL-18, and TNF-α than the sham group (*P <* 0.01; **Figure 5C**). All intervention groups exhibited a significant decrease in these inflammatory cytokine levels compared to the model group (*P <* 0.01; **Figure 5C**). These findings indicate that YXT can reduce serum levels of inflammatory cytokines in HF rat model.

### Effects of YXT on glucose and lipid metabolism in HF rat model

To examine the effect of YXT on myocardial energy metabolism in HF rat models, the ATP content in myocardial tissues was quantified using a micro-method. Serum ATP levels in the model group were significantly decreased compared to those in the sham group (*P <* 0.01; **Figure 6A**). However, all intervention groups exhibited markedly increased serum ATP levels compared to the model group (*P <* 0.01; **Figure 6A**). These results indicate that YXT can enhance myocardial energy metabolism in HF rat models.

**Figure 6:**
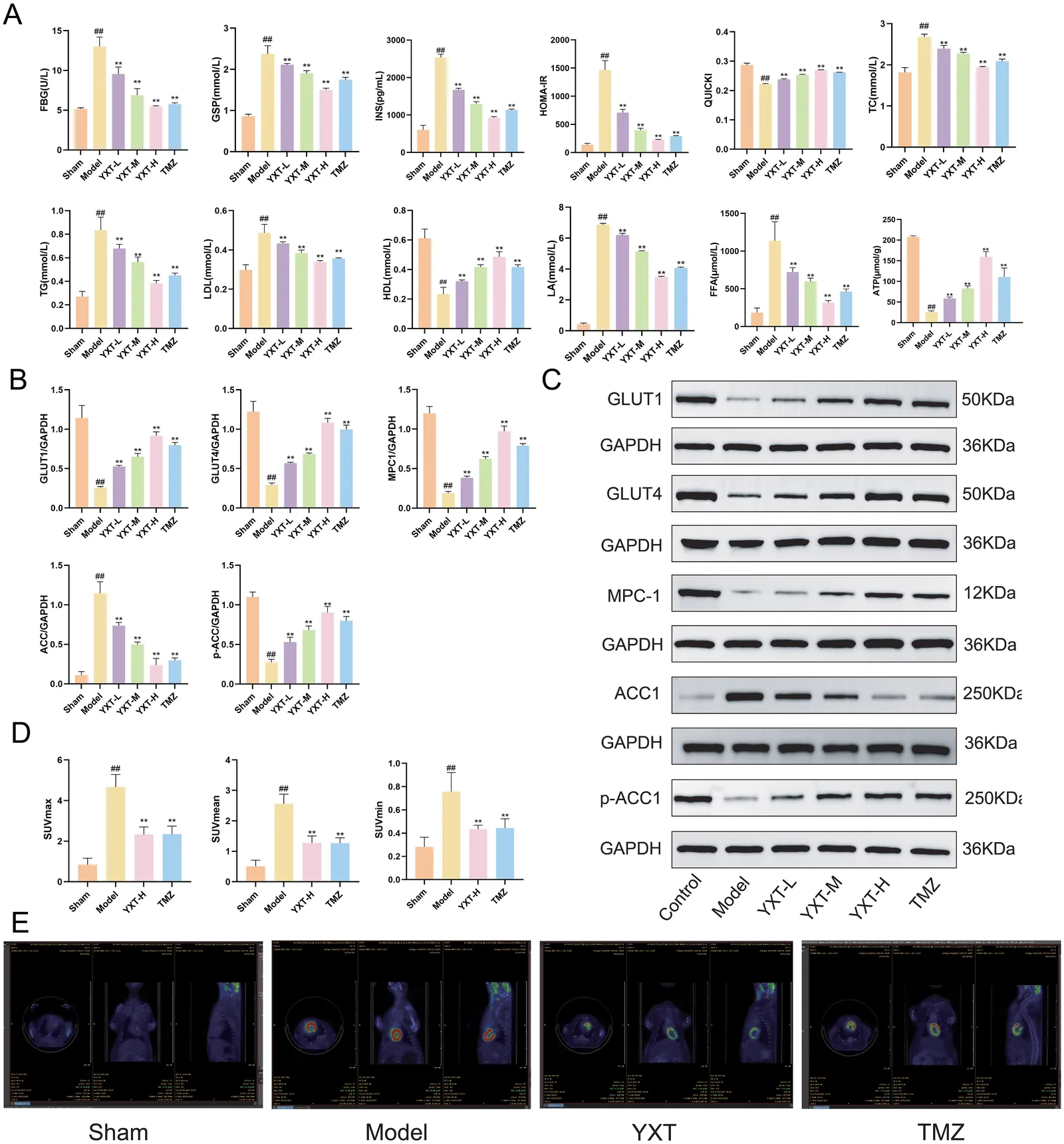
Effects of YXT treatment on glucose and lipid metabolism in HF rat model. **(A)** Expression levels of glucose and lipid metabolism indicators in HF rat model (n=6). **(B, C)** Expression levels of GLUT1, GLUT4, MPC-1, ACC and p-ACC protein(n=3). **(D, E)** The representative cardiac images of ^18^F-FDG PET/CT and the SUV in all groups(n=6). ^##^*P<*0.01 vs. sham group; **P<0.01 vs. model group.

To evaluate the effects of YXT on serum glucose and lipid metabolism in HF rat models, biochemical methods were conducted to measure serum FBG, GSP, triglycerides (TG), CHO, LDL, and HDL. ELISA was utilized to determine serum insulin (INS), lactic acid (LA), and FFA. Furthermore, QUICKI and HOMA-IR were calculated. The model group exhibited significant increases in serum FBG, GSP, HOMA-IR, TC, TG, LDL, INS, LA, and FFA compared to the sham group (*P <* 0.01; **Figure 6A**), whereas QUICKI, HDL and ATP were substantially decreased (*P <* 0.01; **Figure 6A**). Relative to the model group, all intervention groups demonstrated significant reductions in serum FBG, GSP, HOMA-IR, TC, TG, LDL, INS, LA, and FFA (*P <* 0.01; **Figure 6A**), with significant increases in QUICKI,HDL and ATP (*P <* 0.01; **Figure 6A**). These findings indicate that YXT enhances serum glucose and lipid metabolism-related indices in HF rat models.

Subsequently, the effect of YXT on proteins associated with glucose and lipid metabolism was examined. Western blot analysis was performed to detect relevant protein expression in each group. The model group exhibited markedly decreased myocardial GLUT1, GLUT4, MPC-1, and p-ACC expression, and markedly increased ACC expression compared to the sham group (*P <* 0.01; **Figures 6B, C**). All intervention groups exhibited significantly elevated expression of myocardial GLUT1, GLUT4, MPC-1, and p-ACC, and significantly reduced ACC expression compared to the model group (*P <* 0.01; **Figures 6B, C**). These data indicate that YXT activates glucose and lipid metabolism-related proteins, including GLUT1, GLUT4, MPC-1, and p-ACC, and inhibits lipid metabolism-related proteins, such as ACC.

Myocardial glucose metabolism in rats from each group was assessed non-invasively utilizing 18F-FDG PET-CT. Transverse, coronal, and sagittal sections of 18F-FDG uptake among different groups are presented in the PET-CT images. The model group exhibited a significant increase in 18F-FDG uptake compared to the sham group, as evidenced by the intense signals observed in the PET-CT images (**Figures 6D, E**). The SUVmean, SUVmax, and SUVmin of the entire heart in the model group were significantly higher than those in the sham group (*P <* 0.01; **Figures 6D, E**). These findings indicate that YXT ameliorates myocardial glucose metabolism in HF rat models.

### Analysis of heart metabolomics results

#### Central carbon metabolism results

Metabolic effects of YXT on the myocardium of HF rat models were examined using a central carbon metabolomics methodology. The OPLS-DA plot indicated that the data exhibited strong explanatory and predictive capabilities. We identified 49 differentially expressed metabolites between the sham and model groups, among which 46 were upregulated and 3 were downregulated in the model group. However, 49 differentially expressed metabolites were detected between the model and YXT groups, with 46 upregulated and 3 downregulated in the YXT group (**Figures 7A, B**). The heatmap (**Figure 7C**) illustrates the expression changes of differential metabolites among sham, model, and YXT groups, and reveals clustering relationships based on metabolite expression levels. Specifically, the model group exhibited significant increases in fructose-6-phosphate(F6P), fructose-1,6-bisphosphate(FBP), dihydroxyacetone phosphate(DHAP), phosphoenolpyruvic acid, pyruvic acid, 3-phosphoglyceric acid, glycerol-3-phosphate (G3P), fumaric acid and malic acid (*P* < 0.01; **Figures 7D–F**), and elevated levels of glucose-6-phosphate(G6P), LA, sedoheptulose 7-phosphate, ribose 5-phosphate, 6-phosphogluconic acid, α-Ketoglutaric acid and citric acid (*P* < 0.05; **Figures 7D–F**). However, the YXT group exhibited significant reductions in F6P, 3-phosphoglyceric acid, 6-phosphogluconic acid and fumaric acid (*P* < 0.01; **Figures 7D–F**), and decreased levels of G6P.,FBP, DHAP, phosphoenolpyruvic acid, 3-phosphoglyceric acid, G3P, sedoheptulose 7-phosphate, ribose 5-phosphate, citric acid and malic acid (*P* < 0.05; **Figures 7D–F**). YXT can ameliorate alterations in metabolites related to myocardial glycolysis, the TCA cycle, and the pentose phosphate pathway.

**Figure 7:**
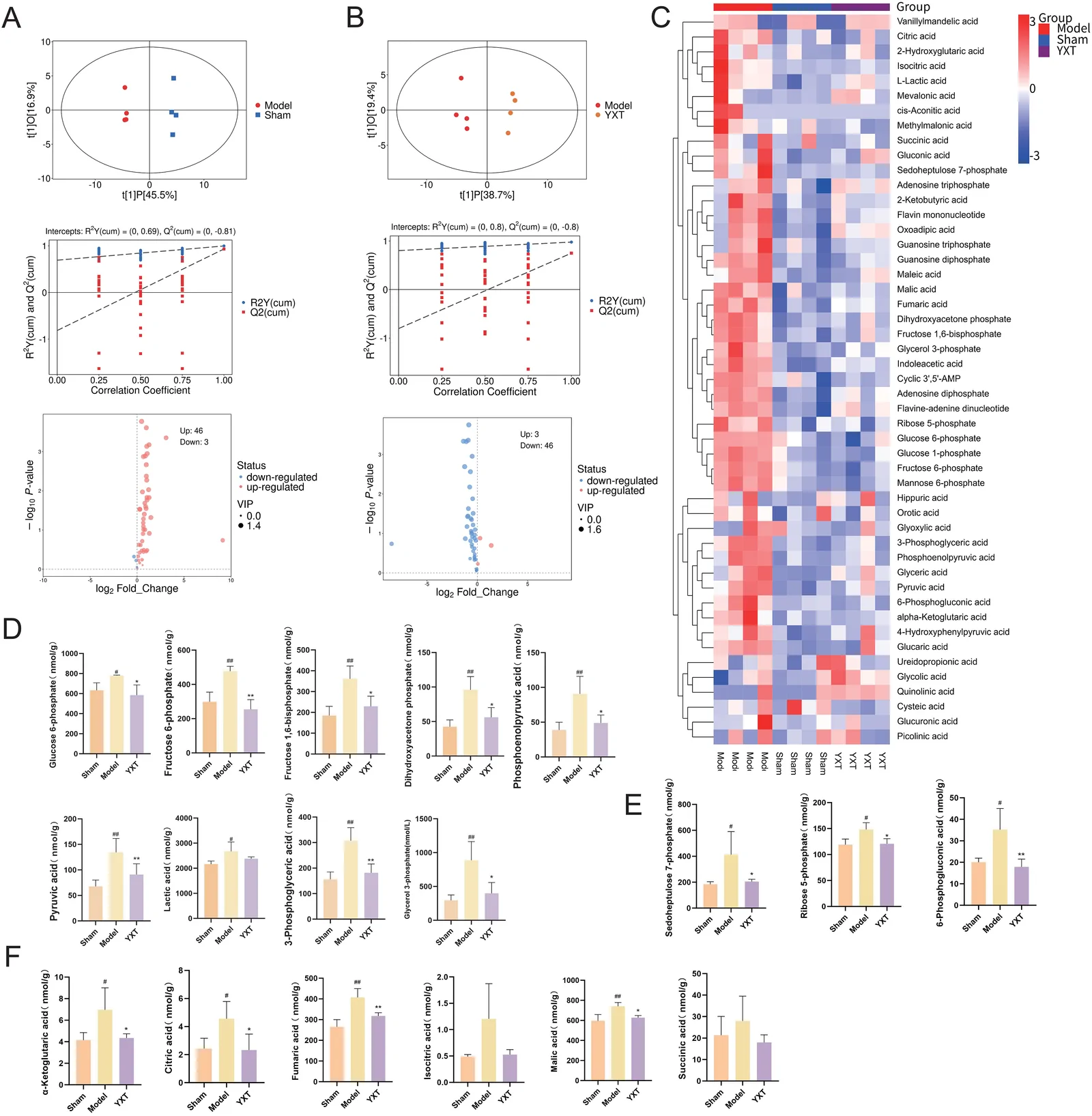
Central carbon metabolism results suggests that YXT treatment improves glucose metabolism pathways in HF rat model. **(A, B)** OPLS-DA S-plot, permutation test result and volcano plot between the sham and model and YXT groups. **(C)** Heatmap of heart central carbon metabolic markers in the sham and model and YXT groups. **(D)** Changes in metabolic substrates related to the glycolysis (n= 4). **(E)** Changes in metabolic substrates related to the pentose phosphate pathway (n=4). ^##^*P* < 0.01 vs. sham group; **P < 0.01 vs. model group. **(F)** Changes in metabolic substrates related to the TCA cycle(n=4). ^##^*P<*0.01 vs. sham group; **P<0.01 vs. model group.

### Quantitative lipidomic results

High-throughput lipid metabolomics was utilized to examine the metabolic effects of YXT on the myocardium of HF rat model. The OPLS-DA plot exhibited strong explanatory and predictive capabilities of the data. We identified 121 differentially abundant metabolites between the sham group and the model group, among which 120 were upregulated and 1 was downregulated in the model group. However, 34 differentially abundant metabolites were identified between model and YXT groups, with the YXT group exhibiting 30 upregulated and 4 downregulated metabolites (**Figures 8A, B**). The heatmap illustrates the expression changes of differential metabolites across sham, model, and YXT groups, highlighting clustering relationships according to metabolite expression levels (**Figure 8C**). Specifically, the model group exhibited significant increases in diacylglycerol (DAG) (18:1/20:4), DAG (18:2/22:6), triacylglycerol (TAG) (48:5/FA18:3), FFA (20:5), FFA (22:3), ceramide (Cer) (18:1/22:1), Hex2Cer (18:1/24:1), Hex2Cer (18:1/26:1), sphingomyelin(SM) (22:0), SM (22:1), lysophosphatidylcholine (LPC) (18:0+AcO), lysophosphatidylethanolamine (LPE) (18:0), lysophosphatidylglycerol (LPG) (18:0), LPG (18:2), bis(monoacylglycerol)phosphate (BMP) (14:0/20:1), phosphatidylcholine (PC) (16:0/18:0 + AcO), PC (18:0/18:3 + AcO), PC (18:2/20:3 + AcO), phosphatidylglycerol (PG) (16:1/18:1), PG (14:0/20:1), PE/O (18:0/22:5), PE (18:0/18:2) and PE (18:2/20:1) (*P* < 0.01; **Figure 8C**), and increases in BMP (18:0/20:2), PI (18:0/22:5), phosphatidylserine (PS) (18:0/18:2), and PS (18:2/22:2) compared with the sham group(*P* < 0.01; **Figure 8C**). The YXT group exhibited significant reductions in DAG (18:2/22:6), FFA (22:3), Cer (18:1/22:1), Hex2Cer (18:1/24:1), Hex2Cer (18:1/26:1), SM (22:0), BMP (14:0/20:1), PC (18:2/20:3+AcO), PG (16:1/18:1), PE/O (18:0/22:5), PG (14:0/20:1), and PS (18:0/18:2) (*P* < 0.01; **Figure 8C**), and decreases in DAG (18:1/20:4), TAG (48:5/FA18:3), FFA (20:5), SM (22:1), LPC (18:0+AcO), LPE (18:0), LPG (18:0), BMP (18:0/20:2), LPG (18:2), PC (16:0/18:0 + AcO), PC (18:0/18:3 + AcO), PI (18:0/22:5), PE (18:0/18:2), PE (18:2/20:1) and PS (18:2/22:2) compared to the model group (*P* < 0.01; **Figure 8C**). YXT reduced the levels of lipid metabolism-related metabolites in HF rat model (**Figure 8C**).

**Figure 8:**
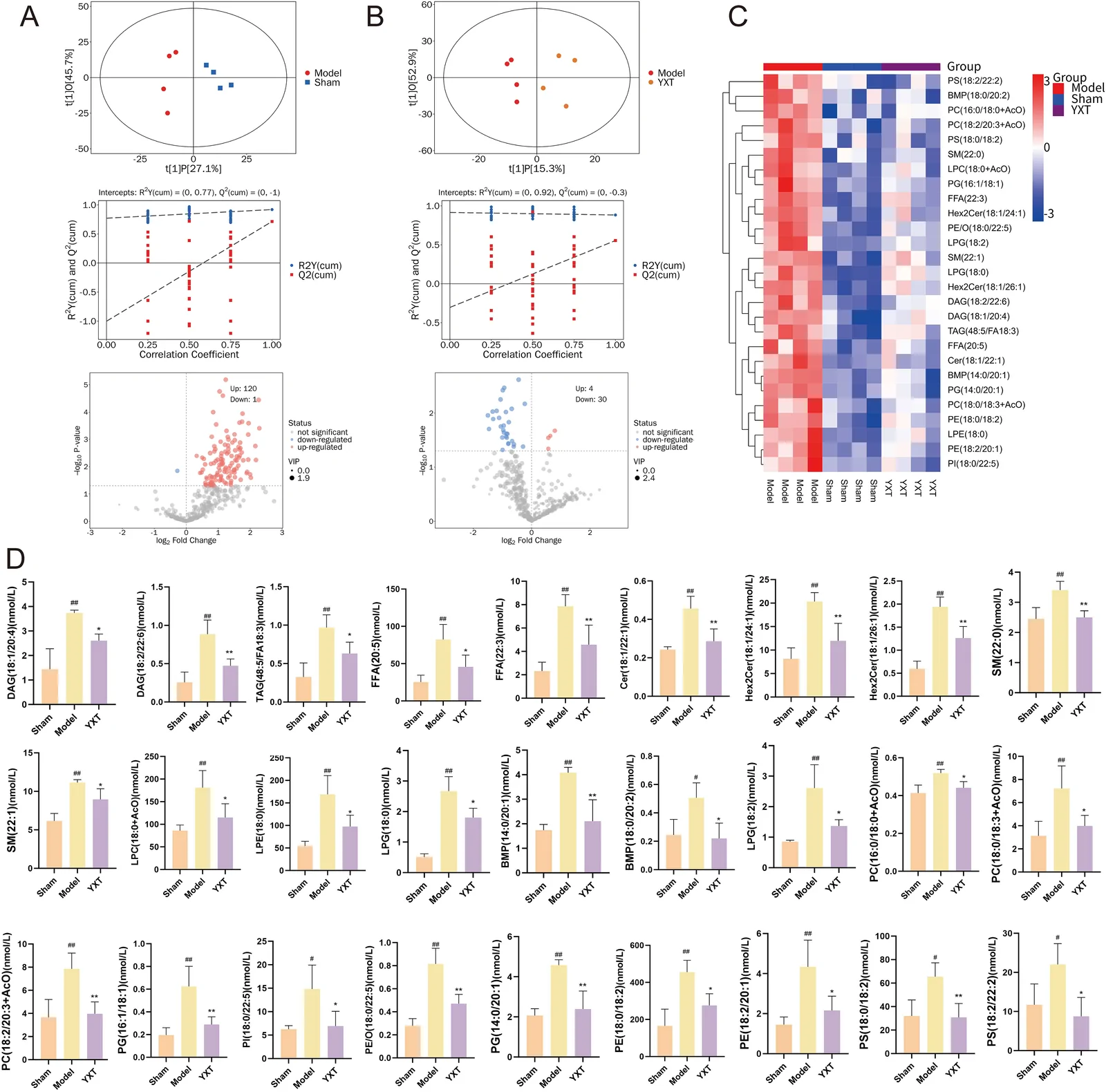
Quantitative lipidomic results suggests that YXT treatment improves lipid metabolism pathways in HF rat model. **(A, B)** OPLS-DA S-plot, permutation test result and volcano plot between the sham and model and YXT groups. **(C)** Heatmap of heart quantitative lipidomic metabolic markers in the sham and model and YXT groups. **(D)** Changes in metabolic substrates related to the Cardiac lipid metabolism (n=4). ^##^*P<*0.01 vs. sham group; **P<0.01 vs. model group.

### Effects of YXT-containing serum on LPS-induced H9c2 cell injury

The CCK-8 assay at 12, 24, and 48 h after LPS intervention in cardiac cells revealed that the optimal time point to achieve the half-maximal inhibitory concentration (IC_50_) was 24 h, and the LPS modeling concentration was established at 25 μg/mL (**Figure 9A**). According to CCK-8 results at 12, 24, and 48 h after intervention with drug-containing serum, the time point for achieving the highest survival rate was 24 h. A concentration of 10% was selected for drug-containing serum and blank serum (**Figure 9B**). Based on the LPS modeling time, drug-containing serum intervention time, and relevant studies (24), TAK-242 and PDTC concentrations were set at 100 μmol/L (*P <* 0.01; **Figure 9C**).

**Figure 9:**
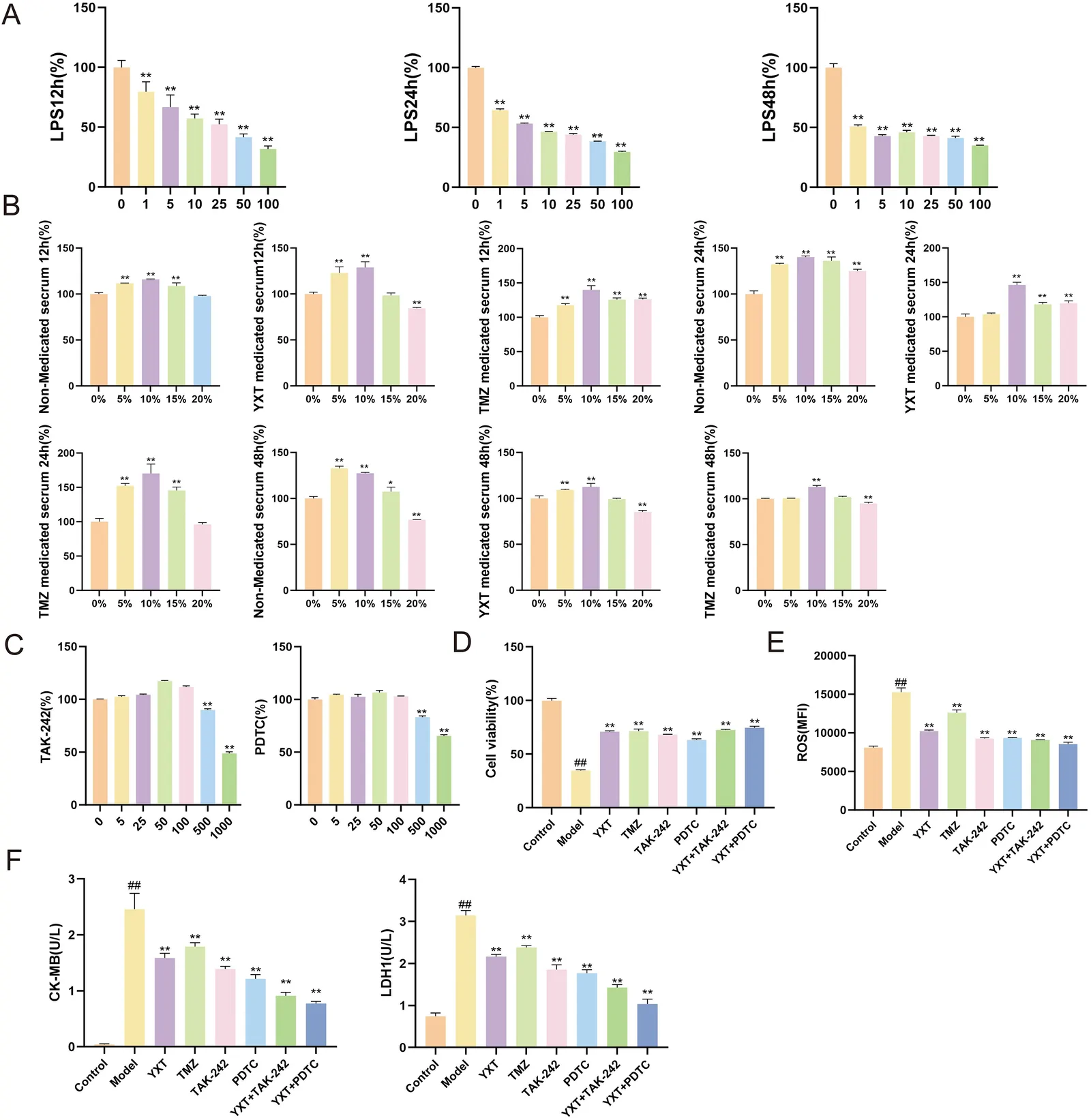
YXT alleviates LPS-induced H9c2 cells injury and improves cell viability. **(A)** Cell viability of H9c2 cells treated by various concentrations and intervention times of LPS(n=3), **P < 0.01 vs. 0μg/mL group; **(B)** Cell viability of H9c2 cells treated by various concentrations and intervention times of serum(n=3), **P < 0.01 vs. 0% group; **(C)** Cell viability of H9c2 cells treated by various concentrations of TAK-242 and PDTC(n=3), **P < 0.01 vs. 100 μmol/L group; **(D)** Effects of YXT on the survival rate of H9c2 cells damaged by LPS(n=3); **(E)** Levels of ROS were measured by flow cytometry (n=3).

The effects of YXT-containing serum on cells in each group were investigated by assessing its impact on the survival rate of H9c2 cardiac cells in the LPS-induced injury model, utilizing the CCK-8 assay. Compared to the control group, the cell survival rate in the model group was substantially decreased (*P <* 0.01; **Figure 9D**). Compared to the model group, the survival rates were significantly enhanced in the YXT-containing serum, TMZ-containing serum, TLR4 inhibitor, NF-κB inhibitor, YXT-containing serum + TLR4 inhibitor, and YXT-containing serum + NF-κB inhibitor groups (*P <* 0.01; **Figure 9D**), indicating that YXT-containing serum can ameliorate the survival rate of LPS-induced H9c2 cardiac cells.

Flow cytometry was utilized to examine the effect of YXT-containing serum on ROS levels in each group. ROS levels in the model group were substantially increased compared to those of the control group(*P <* 0.01; **Figure 9E**). ROS levels were substantially decreased in the YXT-containing serum, TMZ-containing serum, TLR4 inhibitor, NF-κB inhibitor, YXT-containing serum + TLR4 inhibitor, and YXT-containing serum + NF-κB inhibitor groups compared to the model group (*P <* 0.01; **Figure 9E**). These results indicate that YXT-containing serum can reduce ROS levels in the LPS-induced H9c2 cardiac cell injury model.

To assess the effect of YXT-containing serum on LPS-induced injury in H9c2 cardiac cells, the levels of creatine kinase-MB (CK-MB) and lactate dehydrogenase 1 (LDH1) in cardiac cells were quantified. CK-MB and LDH1 levels in H9c2 cardiac cells of the model group were significantly more elevated than those of the control group(*P<*0.01; **Figure 9F**). CK-MB and LDH1 levels in H9c2 cardiac cells were markedly decreased in all intervention groups compared to the normal group (*P<*0.01; **Figure 9F**). Consequently, YXT-containing serum can mitigate LPS-induced H9c2 cardiac cell injury.

### Effects of YXT-containing serum on the LPS/TLR4/NF-κB pathway in LPS-induced H9c2 cells

To evaluate the effect of YXT-containing serum on the LPS/TLR4/NF-κB pathway in LPS-stimulated H9c2 cardiomyocytes, the expression of key pathway components was examined using RT-PCR and Western blot analysis (**Figures 10A, B**). At the mRNA level, expression of TLR4 and MyD88 was significantly higher in the model group than in the control group (*P* < 0.01). In contrast, treatment with YXT-containing serum, TMZ-containing serum, the TLR4 inhibitor, or their combination all led to marked reductions in TLR4 and MyD88 mRNA expression compared with the model group (*P* < 0.01). A similar pattern was observed at the protein level: TLR4 and MyD88 protein expression was markedly elevated in the model group relative to the blank control (*P* < 0.01), and markedly decreased following each of the four interventions (*P* < 0.01).

**Figure 10:**
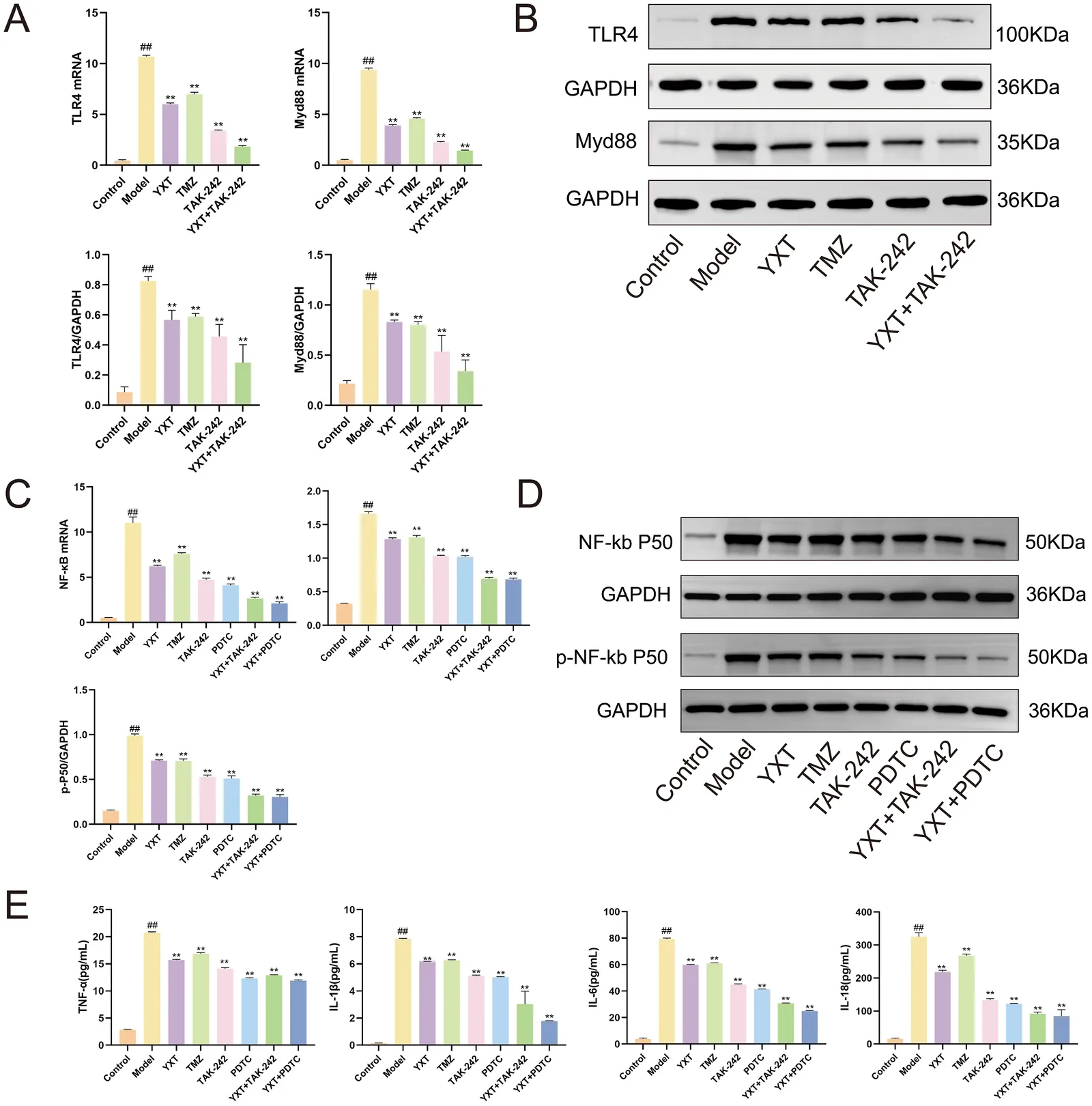
Effects of YXT treatment on the expression levels of the LPS/TLR4/NF-κB signaling pathway in LPS-induced H9c2 cells. **(A–D)** Expression levels of LPS/TLR4/NF-κB pathway gene and protein(n=3). **(C)** Expression levels of inflammatory cytokines(n=6). ^##^*P* < 0.01 vs. control group; **P < 0.01 vs. model group.

The mRNA expression of NF-κB p50 was significantly decreased in the model group than in the control group (*P <* 0.01; **Figures 10B, C**). YXT-containing serum, TMZ-containing serum, TLR4 inhibitor, NF-κB inhibitor, YXT-containing serum + TLR4 inhibitor, and YXT-containing serum + NF-κB inhibitor groups exhibited significantly reduced mRNA expression of NF-κB p50 (*P <* 0.01; **Figures 10B, C**). The protein expression levels of (p-)NF-κB P50 were markedly increased in the model group than in the control group (*P <* 0.01; **Figures 10B, C**). YXT-containing serum, TMZ-containing serum, TLR4 inhibitor, NF-κB inhibitor, YXT-containing serum + TLR4 inhibitor group, and YXT-containing serum + NF-κB inhibitor groups exhibited decreased protein expression of (p-)NF-κB P50 compared to the model group (*P <* 0.01; **Figures 10B, C**).

The LPS/TLR4/NF-κB pathway was activated in LPS-stimulated cardiomyocytes, and YXT inhibited the LPS-induced activation of the LPS/TLR4/NF-κB pathway in H9c2 cardiomyocytes.

To assess the effect of YXT-containing serum on inflammatory factor levels in LPS-induced H9c2 cardiomyocytes, the concentrations of inflammatory cytokines were quantified in each group. The levels of inflammatory factors, including IL-1β, IL-6, IL-18, and TNF-α, were significantly more elevated in the model group than in the control group (*P <* 0.01; **Figure 10E**). All intervention groups exhibited decreased IL-1β, IL-6, IL-18, and TNF-α levels compared to the model group (*P <* 0.01; **Figure 10E**). These results indicate that YXT-containing serum can ameliorate the levels of inflammatory factors in LPS-induced H9c2 cardiomyocytes.

### Effects of YXT-containing serum on glucose and lipid metabolism in LPS-induced H9c2 cells

To assess the effects of YXT-containing serum on energy metabolism in LPS-induced H9c2 cardiomyocytes, ATP levels in each group of cells were measured. The ATP content in H9c2 cardiomyocytes was markedly decreased in the model group compared to the control group (*P <* 0.01; **Figure 11A**). ATP content was markedly increased in each intervention group compared to the model group (*P <* 0.01; **Figure 11A**). These results indicate that YXT-containing serum can enhance the energy metabolism in LPS-induced H9c2 cardiomyocytes.

**Figure 11:**
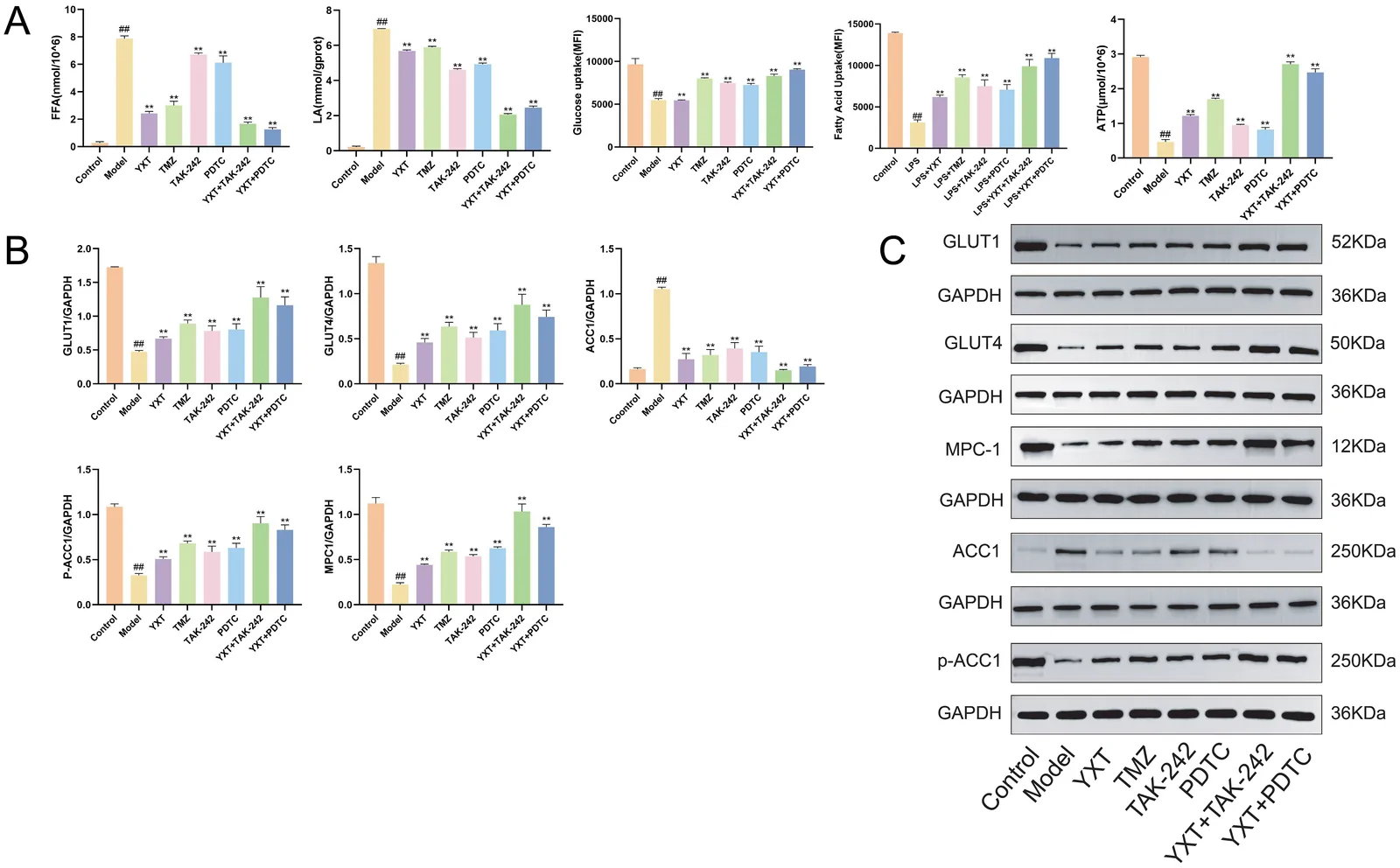
Effects of YXT treatment on glucose and lipid metabolism in LPS-induced H9c2 cells. **(A)** Expression levels of glucose and lipid metabolism indicators in LPS-induced H9c2 cells(n=6). ^##^P<0.01 vs. sham group; **P < 0.01 vs. model group. **(B, C)** Expression levels of GLUT1, GLUT4, MPC-1, ACC and p-ACC protein(n=3). ^##^*P* < 0.01 vs. control group; ^#^*P* < 0.01 vs. control group; **P < 0.01 vs. model group; *P<0.05 vs. model group.

Flow cytometry was utilized to detect the mean fluorescence intensity of glucose uptake and lipid droplet content in each group of cells. The glucose uptake and lipid droplet content in H9c2 cardiomyocytes of the model group was markedly decreased compared to those of the control group (*P <* 0.01; **Figure 11A**). Glucose uptake and lipid droplet content in H9c2 cells of each intervention group was markedly increased compared to those of the model group (*P <* 0.01; **Figure 11A**). These results indicate that YXT-containing serum can enhance glucose uptake and maintained lipid homeostasis to ensure lipid uptake in LPS-induced H9c2 cardiomyocytes.

FFA and LA levels in cardiomyocytes from each group were quantified to evaluate the effects of YXT-containing serum on lipid metabolism-related indicators. FFA and LA levels in H9c2 cardiomyocytes of all other groups were markedly increased compared to those of the control group (*P <* 0.01; **Figure 11A**). FFA and LA levels in H9c2 cardiomyocytes of each intervention group were markedly decreased compared to those of the model group (*P <* 0.01; **Figure 11A**). These findings indicate that YXT-containing serum can ameliorate lipid metabolism in LPS-induced H9c2 cardiomyocytes.

Western blot analysis was utilized to detect the expression of proteins related to glucose and lipid metabolism in cardiomyocytes from each group and to investigate the effect of YXT on glucose and lipid metabolism-related proteins in HF rat models. GLUT1, GLUT4, MPC-1, and p-ACC expression in cardiomyocytes of the model group was considerably decreased, while ACC expression was considerably increased compared to the control group (*P <* 0.01; **Figures 11B, C**). GLUT1, GLUT4, MPC-1, and p-ACC expressions were considerably increased, and ACC expression was significantly decreased in each intervention group compared to the model group (*P <* 0.01; **Figures 11B, C**). These data indicate that YXT-containing serum can activate glucose and lipid metabolism proteins, including GLUT1, GLUT4, MPC-1, and p-ACC, and inhibit lipid metabolism-related proteins, including ACC, in LPS-induced H9c2 cardiomyocytes.

## Discussion

HF, which predominantly manifests in the advanced stages of chronic cardiovascular diseases, is associated with a poor prognosis, high hospitalization rates, and significant mortality rates, imposing a considerable burden on patients, families, and society. Therefore, the active development of pharmacological interventions for HF is of great importance (25). TCM, with its extensive historical background and robust theoretical foundation, plays an increasingly significant role in cardiovascular disease prevention and treatment (26). Recently, TCM has gained international recognition for its distinctive efficacy in treating HF, due to its multi-component, multi-target, and multi-pathway benefits. Furthermore, evidence-based medical research has further substantiated the positive effects of TCM formulations-composed of Chinese herbal medicines—in managing HF (27). Consequently, drug development based on Chinese herbal medicines holds significant potential for HF treatment.

The molecular mechanisms of HF pathogenesis involve mitochondrial dysfunction, cardiac lipotoxicity, maladaptive endoplasmic reticulum (ER) stress, impaired autophagy, cardiac inflammatory responses, apoptosis, endothelial dysfunction, and defective cardiac contractility (28). This study demonstrated that YXT improved cardiac function, reduces myocardial fibrosis, decreases inflammatory cytokine levels, and modulated myocardial energy metabolism-related markers through an integrated approach that includes network pharmacology, molecular docking, and both *in vivo* and *in vitro* experiments. The protective effects were associated with inhibition of LPS/TLR4/NF-κB-associated signaling.

This study utilized UHPLC-MS/MS to identify the active components of YXT. We extracted 1,055 active components from YXT-containing serum, primarily comprising prenol lipids, flavonoids, terpenoids, organooxygen compounds, phenylpropanoids, fatty acyls, steroids, and steroid derivatives. Concurrently, 20 high-confidence components were identified. Following the identification of active components in YXT-containing serum, a network pharmacology analysis was performed to investigate the potential targets of YXT, resulting in the identification of 1,095 targets associated with its effective components. The overlap between the drug targets of YXT and HF-related targets was obtained, yielding 200 intersecting target genes, indicating that YXT may exert therapeutic effects on HF through these 200 targets. Furthermore, GO enrichment analysis revealed that YXT might exert its therapeutic effects on HF through molecular functions, including the MyD88-dependent toll-like receptor signaling pathway, toll-like receptor signaling pathway, glucose metabolic process, positive regulation of NF-kappaB transcription factor activity, and regulation of lipid metabolic process. KEGG enrichment analysis revealed that YXT treats HF through pathways including the NF-kappa B signaling pathway, toll-like receptor signaling pathway, and TNF signaling pathway. PPI network analysis demonstrated that key targets of YXT in treating HF include IL18, IL6, IL1B, and TNF, all of which are associated with the LPS/TLR4/NF-κB pathway. Molecular docking was subsequently performed between the key targets TLR4 and NF-κB and five active components of YXT: astragaloside IV, Ginsenoside_Re, pachymic acid, formononetin, and tanshinone IIb. The findings indicated that all five active components exhibited a favorable binding affinity for the proteins encoded by TLR4 and NF-κB. This study was designed to investigate whether YXT modulates myocardial glucose and lipid metabolism in relation to the LPS/TLR4/NF-κB pathway for HF treatment, integrating findings from network pharmacology, molecular docking, previous research from our group, and literature review.

Inflammation is implicated in HF pathogenesis (29). TLR4 recognizes and binds to LPS, resulting in cellular activation and initiation of an inflammatory response (30). TLR4, an innate immune receptor, belongs to the type I transmembrane glycoprotein receptor family and is significantly associated with the onset, progression, and prognosis of cardiovascular diseases. TLR4 activates the MyD88-dependent signaling pathway by inducing the production of inflammatory factors through the MyD88-dependent signaling pathway. As a downstream signaling component of MyD88, NF-κB p50 is a key transcription factor in inflammatory responses and is integral to the pathogenesis of inflammatory diseases. TLR4 activates the NF-κB pathway, leading to increased levels of inflammatory factors that subsequently induce myocardial fibrosis and contractile dysfunction. This study demonstrates that low-, medium-, and high-dose YXT treatments enhanced cardiac function in HF rat model, mitigated myocardial injury, decreased inflammatory cell infiltration in myocardial tissue, and reduced myocardial fibrosis. The expression of LPS/TLR4/NF-κB signaling components was significantly reduced in low-, medium-, and high-dose YXT groups compared with the model group. Serum levels of inflammatory cytokines, including IL-1β, IL-6, IL-18, and TNF-α, exhibited a significant reduction, demonstrating efficacy similar to that of the TMZ group.

In the inflammatory state of HF, impaired cardiac energetics persist throughout the disease progression, with significant disturbances in glucose and lipid metabolism serving as key pathophysiological changes in HF (31–33). In healthy myocardium, mitochondria are the principal site of energy production in cardiac cells, demonstrating metabolic flexibility by utilizing various energy substrates. FA oxidation in mitochondria accounts for 40%–60% of myocardial energy production, serving as the principal energy source, whereas glucose metabolism accounts for 20%–40%. The remaining energy is sourced from lactate with minor contributions from ketone bodies and amino acid oxidation (9, 34). Following the onset of HF, the heart loses its metabolic flexibility, resulting in diminished ATP production and subsequent energy deficiency (35). This condition is marked by decreased oxygen supply to cardiac cells, prompting a shift in myocardial energy metabolism to satisfy the energy demands of cardiac activity. The hallmark of this shift includes reduced aerobic FA oxidation and enhanced anaerobic glycolysis (12). Moreover, enhanced glycolysis and impaired FA oxidation result in lipid accumulation, which exacerbates myocardial inflammation and worsens HF (36, 37). This study demonstrated that, compared to the model group, low-, medium-, and high-dose YXT treatment increased myocardial ATP levels and enhanced cardiac energetics in HF rat model. Concurrently, YXT reduced serum levels of TC, TG, LDL-C, and HDL-C, thus ameliorating lipid metabolism disorders. Cardiac insulin resistance represents a metabolic disturbance in HF. Mitochondrial dysfunction and the activation of cardiac insulin signaling pathways during HF contribute to myocardial insulin resistance, which reduces insulin-mediated stimulation of glucose uptake and glucose oxidation, resulting in impaired cardiac energetics and exacerbating systolic dysfunction and ventricular remodeling (38). YXT was found to modulate serum FBG, GSP, and insulin levels, along with QUICKI and HOMA-IR, thereby enhancing systemic glucose metabolic disorders and insulin resistance. PET-CT assessed myocardial FDG uptake as an index of glucose uptake. The results demonstrated abnormal glucose accumulation in the peri-infarct zone in the HF model group, whereas the sham group exhibited normal glucose accumulation in the same region. The abnormal glucose accumulation in the peri-infarct area of the model group may be due to the inflammatory response caused by local low energy metabolism in the myocardium (39). After YXT treatment, accumulated glucose was significantly metabolized, yielding results markedly superior to those in the model group and comparable to those obtained with TMZ.

Glucose uptake in cardiac cells is mediated through glucose transporters (GLUTs), primarily involving the most abundant isoforms, GLUT1 and GLUT4. GLUT1 and GLUT4 expression levels primarily determine the rate of glucose transport. During HF progression, pro-inflammatory cytokines (TNF, IL-1β, and IL-6) inhibit glucose transporters (GLUTs), which disrupts glucose uptake and metabolism in cardiac cells (40). The expression levels of GLUT-1 and GLUT-4 in cardiac tissue are decreased (41), resulting in decreased glucose uptake. A compensatory increase in glycolysis occurs simultaneously (42); however, it is insufficient to fully compensate for the energy deficit in HF. The enhanced glycolysis facilitates the conversion of pyruvate to lactate in the cytoplasm, which exacerbates inflammatory responses and ventricular remodeling, thereby further aggravating HF (33, 43). Mitochondrial oxidation of pyruvate (glucose oxidation) plays a crucial role in maintaining normal cardiac structure and function (44). The mitochondrial uptake of pyruvate through the MPC is a crucial regulatory step in glucose oxidation. The inhibition of MPC restricts mitochondrial pyruvate oxidation, which subsequently leads to increased cytosolic lactate accumulation and facilitates the onset of HF. Additionally, elevated glycolytic flux redirects intermediates into the hexosamine biosynthesis pathway (HBP) and PPP. PPP overactivation generates excessive NADPH, which mitigates oxidative damage but disrupts the NADPH/NADP^+^ redox balance, exacerbating mitochondrial oxidative injury (45). Metabolites associated with the TCA cycle are positively correlated with the incidence of HF, a relationship linked to oxidative stress-induced myocardial injury and subsequent ventricular remodeling (45, 46). This study demonstrated that in the model group, the protein expression of GLUT1, GLUT4, and MPC-1 was decreased in rat myocardium. Administration of YXT at low, medium, and high doses, along with TMZ, increased GLUT1, GLUT4, and MPC-1 expression, indicating that YXT enhances myocardial glucose uptake in HF and facilitates pyruvate transport into mitochondria through MPC-1 to participate in the TCA cycle. Central carbon metabolomics indicated increased concentrations of F6P, 3-phosphoglyceric acid, G6P, phosphoenolpyruvic acid, pyruvic acid, LA, dihydroxyacetone phosphate (DHAP), and G3P in the model group. Simultaneously, increased serum LA levels were observed, indicating activation of the glycolytic pathway and PPP in HF rat models. Additionally, TCA cycle metabolites, including citric acid, fumaric acid, and malic acid, were elevated. YXT significantly reduced the levels of glycolytic metabolites, including F6P, 3-phosphoglyceric acid, G6P, phosphoenolpyruvic acid, pyruvic acid, DHAP, and G3P, suggesting an altered glycolytic metabolite profile in HF rat models. Furthermore, YXT reduced PPP-related metabolite levels, consistent with modulation of the PPP, including G6P and 6-phosphogluconic acid, thereby mitigating mitochondrial redox injury. YXT reduced myocardial TCA cycle metabolite citric acid levels in HF rat models, indicating its involvement in modulating the TCA cycle to alleviate mitochondrial oxidative stress damage. These changes suggest that YXT may modulate glucose-related metabolic pathways, including glycolysis, the TCA cycle, and the pentose phosphate pathway.

The rate of myocardial FA oxidation decreases with the progression of HF severity; however, FA uptake into the cytoplasm remains constant. Lipid accumulation within the heart leads to lipotoxicity. These lipids lead to mitochondrial dysfunction in cardiomyocytes (47), which results in enhanced mitochondrial fission, decreased fusion, induction of insulin resistance, and myocardial fibrosis, thereby exacerbating HF (48). Acetyl-CoA carboxylase (ACC) is a rate-limiting enzyme involved in *de novo* FA synthesis, catalyzing the conversion of acetyl-CoA to malonyl-CoA and thereby enhancing FA synthesis (49). ACC phosphorylation inhibits its activity, resulting in increased mitochondrial FA oxidation and enhanced insulin sensitivity in cardiomyocytes. In the model group, p-ACC expression was decreased, whereas ACC expression was increased (50). Treatment with low-, medium-, and high-dose YXT, along with TMZ, increased p-ACC expression and reduced ACC expression, and were associated with altered lipid metabolic markers in HF rat models. Moreover, serum levels of FFAs were elevated in the model group, consistent with existing clinical studies that report increased circulating FA levels in patients with HF (51, 52). Simultaneously, myocardial lipid metabolomics revealed increased levels of FFA(20:5) and FFA(22:3), suggesting increased myocardial and circulating FFA content in rats with HF. YXT reduced myocardial and circulating FFA levels, promoted myocardial FA oxidation, and decreased lipid accumulation.

Moreover, current studies have demonstrated the existence of various lipid disorders in failing myocardium (53). The accumulation of sphingolipids can induce inflammatory responses and lipid deposition in cardiac cells, resulting in myocardial fibrosis, ventricular remodeling, and energy metabolism disorders, which contribute to HF (54–56). Current evidence suggests that DAG, TAG, glycerophospholipids (PC, PE, PG, PS, and PI), and their metabolites (LPC, LPE, and LPG), and sphingolipids (SM, Cer, and HexCer) are excessively accumulated in myocardial tissues of HF (57, 58). In model groups, BMP, PC, PE, PG, LPC, and SM were increased, whereas the YXT intervention mitigated lipid disturbances, including those of DAG, TAG, glycerophospholipids, and sphingolipids. YXT was associated with changes in myocardial lipid profiles, including FA-, glycerophospholipid-, and sphingolipid-related pathways.

*In vitro* experiments utilized an LPS-induced H9c2 cell injury model as the research subject. H9c2(2-1) cells represent a subclone of the original clonal cell line established from embryonic BD1X rat heart tissue by B. Kimes and B. Brandt and are extensively utilized in toxicology and cardiovascular disease research. LPS, a major endotoxin derived from Gram-negative bacteria, is an inflammatory inducer extensively employed to establish myocardial injury models in both *ex vivo* and *in vivo* settings. LPS induces cardiac cell injury, resulting in increased lactate dehydrogenase (LDH), creatine kinase (CK), and creatine kinase-MB (CK-MB) levels (59, 60), and stimulates the release of inflammatory cytokines, including IL-1β, IL-6, IL-18, and TNF-α (61, 62). Meanwhile, HF is characterized by changes in myocardial redox regulation, primarily marked by oxidative stress (63). LPS induces the secretion of inflammatory mediators and increases ROS levels, thereby exacerbating oxidative damage in H9c2 cells (64, 65). LPS can induce energy metabolism disorders in H9c2 cells, potentially by enhancing glycolysis and suppressing oxidative phosphorylation, leading to dysregulated myocardial glucose metabolism (66). LPS stimulation concurrently impairs FA oxidation in H9c2 cells, resulting in lipid metabolism disorders (67). Consequently, the LPS-induced H9c2 cardiac cell injury model effectively recapitulates the pathophysiological alterations associated with inflammatory responses and energy metabolic disorders following HF. Furthermore, this model effectively evaluates the effects of YXT on the LPS/TLR4/NF-κB pathway, aligning closely with the experimental objectives. This study demonstrated that LPS treatment decreased H9c2 cell viability, upregulated inflammatory protein expression, increased ROS levels, and elevated LDH and CK-MB levels, confirming the effective establishment of the model. Subsequently, the YXT-containing serum group significantly inhibited the expression of LPS/TLR4/NF-κB pathway. The concentrations of inflammatory cytokines, including IL-1β, IL-6, IL-18, and TNF-α, in the cell supernatant, and intracellular ROS levels, were markedly reduced. Decreased levels of these inflammatory cytokines and intracellular ROS were similarly observed in the TLR4 inhibitor, NF-κB inhibitor, YXT-containing serum + TLR4 inhibitor, and YXT-containing serum + NF-κB inhibitor groups. Among these, the YXT-containing serum + TLR4 inhibitor and YXT-containing serum + NF-κB inhibitor groups exhibited the most significant effects.

In LPS-induced myocardial injury, the cardiac uptake of energy substrates (FFA and glucose) is frequently reduced (68). In this study, the model group exhibited decreased glucose uptake and lipid droplet content as determined by 2-NBDG and BODIPY 493/503 assays, along with reduced ATP levels compared with the control group, a phenomenon also observed in other studies (69, 70). Simultaneously, the model group exhibited reduced expression of glucose and lipid metabolism-related proteins, including GLUT1, GLUT4, MPC-1, and p-ACC, while the expression of lipid metabolism-related proteins, including ACC, was increased, alongside increased levels of FFA and LA. Both YXT-containing serum and TMZ-containing serum ameliorated glucose uptake and lipid droplet content in LPS-treated H9c2 cardiomyocytes, decreased FFA and LA levels, and enhanced the expression of glucose and lipid metabolism-related proteins. Furthermore, cardiomyocytes in the TLR4 inhibitor group and NF-κB inhibitor group also exhibited improvements in glucose uptake and lipid droplet content, FFA and LA levels, and expression of glucose and lipid metabolism-related proteins compared with the model group. The most significant therapeutic effects were observed in the YXT-containing serum + TLR4 inhibitor group and the YXT-containing serum + NF-κB inhibitor group, and these effects may be associated with inhibition of LPS/TLR4/NF-κB-associated signaling.

This study employed TMZ as a positive control. TMZ is an extensively utilized anti-anginal drug that alters energy metabolism from long-chain FAs to glucose by inhibiting 3-ketoacyl-CoA thiolase (KAT). This mechanism decreases FA oxidation and promotes glucose oxidation in ischemic heart disease. This results in decreased myocardial oxygen consumption and improved cardiac function, serving as a partial FA oxidation inhibitor. TMZ enhances glucose oxidation and decreases lactate production through the modulation of PDH activity (71). Clinical and basic research have confirmed its efficacy in treating HF (72, 73). Our results demonstrate that TMZ enhances cardiac function, reduces myocardial fibrosis, modulates the expression of LPS/TLR4/NF-κB signaling pathway in myocardial tissue (74), lowers serum inflammatory marker levels (75), improves myocardial energy metabolism, and improves glucose and lipid metabolite levels in HF rat models, which is consistent with other research findings (76–79). Compared with YXT, TMZ exhibited similar efficacy in enhancing inflammatory factors, myocardial fibrosis, and myocardial energy metabolism levels. This may be due to the complex composition and multiple targets of YXT, which enabled modulation of various signaling pathways.

### Limitations

This study has several limitations. Firstly, although co-treatment with TLR4 or NF-κB inhibitors further enhanced the effects of YXT, the present study does not establish that TLR4/NF-κB suppression directly mediates the metabolic effects of YXT, and direct glycolytic or fatty acid oxidation flux was not measured. In addition, the involvement of alternative metabolic regulators such as AMPK/ACC and PI3K/AKT was not assessed; and pharmacokinetic exposure and a formal head-to-head equivalence comparison with trimetazidine were not performed. Future studies should therefore use genetic knockdown or knockout, rescue experiments, and non-additive epistasis analyses with TAK-242/PDTC to test pathway dependence; measure metabolic flux using Seahorse analysis, 13C-labeled substrate tracing, or BODIPY FL C16 assays; assess AMPK/ACC and PI3K/AKT signaling and mitochondrial function; examine MD2/TLR4 complex formation, MyD88/TRIF branching, p65 nuclear translocation kinetics, and direct binding by biophysical assays; test defined purified constituents; test defined purified constituents and perform pharmacokinetic and head-to-head comparative studies with trimetazidine. Second, although YXT exhibited a trend toward reducing specific glucose and lipid metabolites, the changes lacked statistical significance, potentially due to the limited sample size. Future studies with extensive sample sizes are required to clarify the therapeutic efficacy of YXT. Thirdly, significant differences exist between rats and humans regarding coronary collateral circulation, metabolic rate, and pharmacokinetics, this discrepancy may constrain how directly these findings translate to human patients. At the same time, it must be acknowledged that LPS-treated H9c2 cells reproduce selected inflammatory features of cardiac injury, rather than the full pathophysiology of ischemic heart failure. Future investigations incorporating these factors will further clarify how YXT exerts its preventive and therapeutic effects against IHF.

## Conclusions

To explore the potential active constituents and mechanisms of YXT against IHF, we combined network pharmacology, molecular docking, and *in vivo* and *in vitro* experiments. Our results show that YXT improved cardiac function, reduced myocardial fibrosis, and attenuated ultrastructural damage in IHF rats. These beneficial effects were accompanied by inhibition of LPS/TLR4/NF-κB-associated signaling, reduced inflammatory cytokine levels, and altered glucose/lipid metabolic profiles. Notably, co-treatment with TLR4 or NF-κB inhibitors further enhanced these effects, supporting the involvement of this pathway in the actions of YXT. Overall, this study provides preliminary evidence supporting further investigation of YXT and TCM-derived approaches for IHF.

## Data Availability

The original contributions presented in the study are included in the article/**Supplementary Material**. Further inquiries can be directed to the corresponding authors.

## Glossary

ACC: Acetyl-CoA carboxylase
ANP: Atrial Natriuretic Peptide
ATP: adenosine triphosphate
BNP: Brain Natriuretic Peptide
CCK-8: Counting Kit 8
Cer: ceramide
CK: creatine kinase
CK-MB: creatine kinase-MB
DAG: diacylglycerol
DHAP: dihydroxyacetone phosphate
DHAP: dihydroxyacetone phosphate
EF: ejection fraction
ELISA: enzyme-linked immunosorbent assay
ER: endoplasmic reticulum
ESI: electrospray ionization
F6P: fructose-6-phosphate
FAs: fatty acids
FBG: fasting blood glucose
FBP: fructose-1,6-bisphosphate
FDG: fluorodeoxyglucose
FFA: free FA
Fins: fasting insulin
FS: fractional shortening
G3P: glycerol-3-phosphate
G3PP: G3P phosphatase
G6P: glucose-6-phosphate
G6PD: glucose-6-phosphate dehydrogenase
GA3P: glyceraldehyde-3-phosphate
GAPDH: glyceraldehyde-3-phosphate dehydrogenase
GK: glucokinase
GlcN-6-P: glucosamine-6-phosphate
GLUTs: glucose transporters
GO: gene ontology
GPAT: glycerol-3-phosphate acyltransferase
GPD1: glycerol-3-phosphate dehydrogenase 1
GSP: glycated serum protein
HPLC: High-Performance Liquid Chromatography
H&E: hematoxylin and eosin
HBP: hexosamine biosynthesis pathway
HDL-C: high-density lipoprotein cholesterol
HF: Heart failure
HOMA-IR: homeostasis model assessment of insulin resistance
IC: inhibitory concentration
IDA: information-dependent acquisition
IHF: ischemic heart failure
IL-18: Interleukin 18
IL-1β: interleukin-1β
IL-6: interleukin 6
INS: insulin
IS: internal standard
KAT: 3-ketoacyl-CoA thiolase
KEGG: kyoto encyclopedia of genes and genomes
LA: lactic acid
LBP: Lipopolysaccharide Binding Protein
LDH: lactate dehydrogenas
LDH1: lactate dehydrogenase 1
LDL-C: low-density lipoprotein cholesterol
LPA: lysophosphatidic acid
LPC: lysophosphatidylcholine
LPE: lysophosphatidylethanolamine
LPG: lysophosphatidylglycerol
LPS: lipopolysaccharide
LVEF: left ventricular ejection fraction
LVIDd: left ventricular end-diastolic diameter
LVIDs: left ventricular end-systolic diameter
MPC: mitochondrial pyruvate carrier
MTBE: methyl tert-butyl ether
MyD88: myeloid differentiation primary response 88
NT-proBNP: N-Terminal Pro-Brain Natriuretic Peptide
PA: phosphatidic acid
PBS: phosphate-buffered saline
PC: phosphatidylcholine
PE: phosphatidylethanolamine
PET-CT: positron emission tomography–computed tomograph
PFK-1: phosphofructokinase-1
PG: phosphatidylglycerol
PI: Phosphatidylinositol
PKM2: pyruvate kinase muscle isozyme M2
PLA2: Phospholipase A2
PLs: phospholipases
PPI: protein-protein interaction
PPP: pentose phosphate pathway
PS: phosphatidylserine
QUICKI: quantitative insulin sensitivity check index
ROI: the region of interest
RT-qPCR: reverse transcription quantitative polymerase chain reaction
SM: sphingomyelin
SPF: specific pathogen-free
SUV: standardized uptake value
TAG: triacylglycerol
TBST: tris-buffered saline
TC: total cholesterol
TCA: the tricarboxylic acid
TCM: traditional Chinese medicine
TG: triglyceride
TLR4: toll-like receptor 4
TMZ: trimetazidine
TNF-α: tumor necrosis factor-α
UHPLC-MS/MS: ultra-high-performance liquid chromatography coupled with mass spectrometry
YXT: Yixintai granules
