# Supplementary material for: Yixintai granules enhance glucose and lipid metabolism after ischemic heart failure by regulating inflammatory factors via the LPS/TLR4/NF-κB signaling pathway
Source: Front Immunol. 2026 Oct 16;17:1984202. doi: 10.3389/fimmu.2026.1984202 (PMC13719404; doi:10.3389/fimmu.2026.1984202)
Supplement: Supplementary file 1 [file DataSheet1.pdf]

frontiers

The International Society for the Advancement of Cytometry (ISAC) has highlighted the importance of including comprehensive methodological information to ensure data reproducibility and reliability. In line with this, Frontiers in Immunology now requires authors to submit a checklist for manuscripts that involve flow or mass cytometry. This checklist helps standardize the reporting process, improving the quality and transparency of published data. By doing so, we support scientific progress, making it easier for other researchers to replicate and validate experiments.

This form should be submitted with any manuscripts using flow or mass cytometry.

Sample/specimen/material description

- [ ] Total blood
- [ ] PBMCs
- [x] Organ digests

Other____

Did the samples suffer any treatment before or after incubation with the antibodies?

- [ ] Drug ____________________________________
- [ ] Cell permeabilization ______________________
- [ ] Dye _____________________________________
- [ ] Propidium iodine
- [x] Not applicable

Other ____

Instrument and antibodies

Name of the Cytometer Beckman A00-1-1102

Antibodies and targets	Fluorochrome/ Metal	Catalog number/Company
e.g. anti-CD4	FITC	Cat. XXX/ XXX ltd.
	BODIPY493/503 methyl bromide	HY-D1614/MCE

	2-NBDG	HY-116215/MCE
	ROS	S0033S/beyotime

Data analyses

1. Name of the software CytExpert________________________________
2. Reference gating strategy in the manuscript or supplementary material

Gating strategy in (eg Figure X) Supplementary file1______________________________
